# Restoration of CPT1A-mediated fatty acid oxidation in mesothelial cells protects against peritoneal fibrosis

**DOI:** 10.7150/thno.84921

**Published:** 2023-08-15

**Authors:** Wenyan Su, Zuoyu Hu, Xiaohong Zhong, Ansheng Cong, Ying Zhang, Zhanmei Zhou, Jianyi Li, Cailing Su, Yujie Huang, Wei Cao

**Affiliations:** 1 Division of Nephrology, Nanfang Hospital, Southern Medical University, State Key Laboratory of Organ Failure Research, Guangdong Provincial Key Laboratory of Nephrology, 1838 North Guangzhou Ave, Guangzhou 510515, P. R. China; 2 Division of Nephrology, The Second Affiliated Hospital of Guangzhou Medical University, Changgang East Road, Guangzhou 510260, P.R. China; 3 Department of Urology, The First Affiliated Hospital of Shandong First Medical University & Shandong Provincial Qianfoshan Hospital, Shandong medicine and Health Key Laboratory of Organ Transplantation and Nephrosis, Shandong Institute of Nephrology, Jinan 250013, P.R. China

**Keywords:** peritoneal fibrosis, mesothelial cell, fatty acid oxidation, carnitine palmitoyltransferase 1A, peritoneal dialysis

## Abstract

**Background:** Peritoneal dialysis (PD) is limited by gradual fibrotic remodeling in the peritoneum, a process involving profibrotic response of mesothelial cells. However, the role of fatty acid oxidation (FAO) and carnitine palmitoyltransferase 1A (CPT1A) in this process remains unexplored.

**Methods:** FAO and CPT1A expression were characterized in mesothelial cells from patients on long-term PD and from a mouse model of PD using multiple experimental methods, including single-cell sequencing, seahorse assay, real-time quantitative PCR, Western blot, and immunofluorescence staining. Overexpression of CPT1A was achieved in a human mesothelial cell line and in primary mouse mesothelial cells. Finally, genetic and pharmacological manipulations of CPT1A were performed in a mouse model of PD.

**Results:** Herein, FAO and CPT1A expression were reduced in mesothelial cells from patients on long-term PD, which negatively correlated with expression of fibrogenic markers in these cells. This was corroborated in PD mice, as well as in mouse and human mesothelial cells incubated with transforming growth factor (TGF) β1. CPT1A overexpression in mesothelial cells, which prevented TGFβ1-induced suppression of mitochondrial respiration, restored cellular ATP levels and downregulated the expression of fibrogenic markers. Furthermore, restoration of FAO by overexpressing CPT1A in PD mice reversed profibrotic phenotype in mesothelial cells and reduced fibrotic lesions in the peritoneum. Treatment with the CPT1A activator C75 induced similar therapeutic benefit in PD mice. In contrast, inhibition of FAO with a CPT1 inhibitor caused more severe fibrosis in PD mice.

**Conclusions:** A defective FAO is responsible for the profibrotic response of mesothelial cells and thus the peritoneal fibrogenesis. This aberrant metabolic state could be improved by modulating CPT1A in mesothelial cells, suggesting FAO enhancement in mesothelial cells is a potential treatment of peritoneal fibrosis.

## Introduction

Peritoneal dialysis (PD) remains the leading form of home-based dialysis for the treatment of end-stage kidney disease 1,2. Efficiency of PD depends on a healthy and functional peritoneal membrane 3. Unfortunately, long-term exposure of the peritoneum to bioincompatible, glucose-based dialysis solutions often results in progressive fibrotic lesions, characterized by enhanced production of profibrotic factors and excessive deposition of extracellular matrix. This development of peritoneal fibrosis leads to a gradual increase in solute transport rate and loss of ultrafiltration, culminating in PD failure that affects up to 50% of patients undergoing PD 4,5. Cellular mechanisms facilitating this fibrogenic response are limited, and thus strategies to improve outcomes of patients on PD are lacking.

Mesothelial cells are among the most common cells in the resting peritoneum 6. They are plastic and can acquire a profibrotic phenotype in response to extended PD, contributing to development of peritoneal fibrosis 2,6,7. While numerous genetic and humoral factors regulating this profibrotic response have been identified 6,8-11, much less is known about the role of metabolism. Si *et al.* recently reported that mesothelial cells, when subjected to long-term PD, develop a hyperglycolytic metabolism to fuel the overproduction of extracellular matrix, linking energy production to the altered mesothelial cell phenotype 6. Fatty acid oxidation (FAO) in many cell types has been implicated in ATP generation and scavenging of reactive oxygen species during cellular stress 12-15. However, the role and importance of FAO in mesothelial cells in the progression of peritoneal fibrosis remain undefined.

A rate-limiting step of FAO is the shuttling of long-chain fatty acids into mitochondria by carnitine palmitoyltransferase 1 (CPT1). Of the CPT1 family, CPT1A is the most widely distributed isoform and has high catalytic activity 16. Reduced expression of CPT1A in tubular cells has been implicated in kidney fibrosis in animal models of kidney injury 12,17. However, the expression and the potential role of CPT1 in mesothelial cells in the setting of PD have not been examined.

Hence, in the context of PD, we investigate the role of FAO in mesothelial cells during peritoneal fibrosis and assess whether targeting FAO reduces PD-related peritoneal fibrosis. We address these issues using human single-cell RNA sequencing (scRNA-seq) data, *in vivo* mouse models and *in vitro* cell models.

## Materials and Methods

### Human samples

Protocols using human samples were approved by the Ethics Committee of the Second Affiliated Hospital of Guangzhou Medical University (2021-hs-61), and were performed according to the Declaration of Helsinki.

#### Isolation of human peritoneal mesothelial cells by flow cytometry

For isolating mesothelial cells from patients on long-term PD, PD effluents were obtained from 12 patients who were diagnosed with end-stage kidney disease and received PD for more than 6 years (8 females, 4 males; age, 48 ± 12.4 years). The fluid of the whole PD efferent bag was centrifuged. Then, the cell pellet was washed and resuspended in PBS containing 0.5% BSA 6. Mesothelial cells were isolated by fluorescence-activated cell sorting using UPK3B antibody 6.

For isolation of normal human mesothelial cells, omentum specimens were obtained from 6 nonuremic patients without known cancer, undergoing elective abdominal surgery (4 females, 2 males; age, 53 ± 7.9 years). Tissues were cut into about 4-mm^2^ pieces, digested with 0.25% Trypsin-EDTA and centrifuged at 500g for 5 min. Then, the cell pellet was washed and resuspended in PBS containing 0.5% BSA 6. Mesothelial cells were isolated by fluorescence-activated cell sorting using UPK3B antibody 6.

#### Single-cell Analysis

A scRNA-seq data of human peritoneal cells in the GEO database (GSE130888) was reanalyzed to investigate the metabolic alterations of human mesothelial cells in response to long-term PD. Cells were grouped and annotated by a marker gene set from a previous study (Table S1) 6. Gene expression in cells was analyzed using the Seurat package. Gene expression data was mapped on gene ontology pathways using ClusterProfiler package. Cell trajectory analysis was performed using Monocle3 package.

### Experimental PD model

All animal procedures were approved by the Animal Ethics Committee of Nanfang Hospital (IACUC-LAC-20220711-002) and all procedures were done in accordance with their guidelines. Male C57BL/6 mice (8 weeks old) weighing 20-24 g were obtained from the Institutional Animal Experiment Center. Mice were maintained in a temperature-controlled room with a 12-hr light-dark cycle (lights on at 8:00 a.m.) and were allowed free access to food and pure water.

The mouse PD model of peritoneal fibrosis was induced by daily intraperitoneal injection of 4.25% PD fluid (Dianeal containing 4.25% glucose; Baxter Healthcare, IL, USA) at 100mL/kg body weight for 6 weeks. Sham-operated mice were subject to daily intraperitoneal injection of saline for 6 weeks.

To improve FAO in mesothelial cells in PD mice, C75 was injected intraperitoneally three times per week (5 mg/kg body weight; Selleckchem, Houston, TX, USA) from the first day of PD fluid administration. The C75 treatment did not affect the body weight and the serum levels of creatinine, alanine aminotransferase and aspartate aminotransferase in PD mice (Figure S8F).

To pharmacological blockade of FAO in mesothelial cells in PD mice, etomoxir was injected intraperitoneally three times per week (30 mg/kg body weight; Selleckchem) from the first day of PD fluid administration.

### Gene Delivery in Animals

The full-length mouse CPT1A cDNA was inserted into a CMV-MCS-2A-GFP vector. After sequencing ensured accuracy of the vector, adeno-associated virus (AAV serotype 9) was packaged, purified, and titrated by GeneChem (Shanghai, China). AAV9 harboring either the CPT1A (AAV-CPT1A) or the control sequence (AAV-NC) was intraperitoneally injected into the mice at 5 × 10^11^ v.g. three weeks before PD fluid administration.

### Cell cultures

#### Culture of primary mouse peritoneal mesothelial cells

Cultures of primary mouse peritoneal mesothelial cells were derived from mesentery resected from 8-week-old male C57BL/6 mice 18. Concisely, mesenteric tissues were obtained from mice under anesthesia with intraperitoneal (i.p.) injection of sodium pentobarbital (50 mg/kg body weight), and then digested with 0.25% Trypsin-EDTA for 10 min at 37 °C with gentle rotation. Next, the digested solution was triturated in growth medium (Dulbecco's Modified Eagle Medium with 15% FBS), centrifuged, and seeded onto a 60mm culture dish. Purity of cultured mesothelial cells was confirmed by morphological and immunofluorescence analysis, showing selective expression of mesothelial cell-enriched markers cytokeratin-18 and vimentin but not (minimally) of markers of endothelial cells (CD31) and leukocytes (CD45). Cells of passage 1 and 2 were used for the experiments.

#### Culture of human mesothelial cells

Mesothelial cells (Met-5A, human; American Type Culture Collection, VA, USA) were cultured in Medium 199 supplemented with 10% FBS, 1% Insulin-Transferrin-Selenium, and 1% penicillin-streptomycin (all from Gibco, Grand Island, NY, USA) at 37 °C in 5% CO_2_ atmosphere.

### Statistical analysis

Quantitative data was expressed as means and standard deviations or as the median with interquartile range. Power analyses for independent *t*-tests were used to assess the number of animals required (nQuery version 8.2.1, Statistical Solutions Ltd., Ireland). Normality tests were assessed by Shapiro-Wilk statistics. Differences among groups were determined by one-way ANOVA or unpaired *t* test with Bonferroni correction for multiple testing. When normality was rejected, the nonparametric Kruskal-Wallis test was employed. A *P* value of less than 0.05 was considered statistically significant. All analyses were conducted with SPSS 20.0 for Windows (SPSS, Inc., Chicago, IL, USA).

## Results

### Reduction of FAO in mesothelial cells characterizes patients on long-term PD

**Figure 1:**
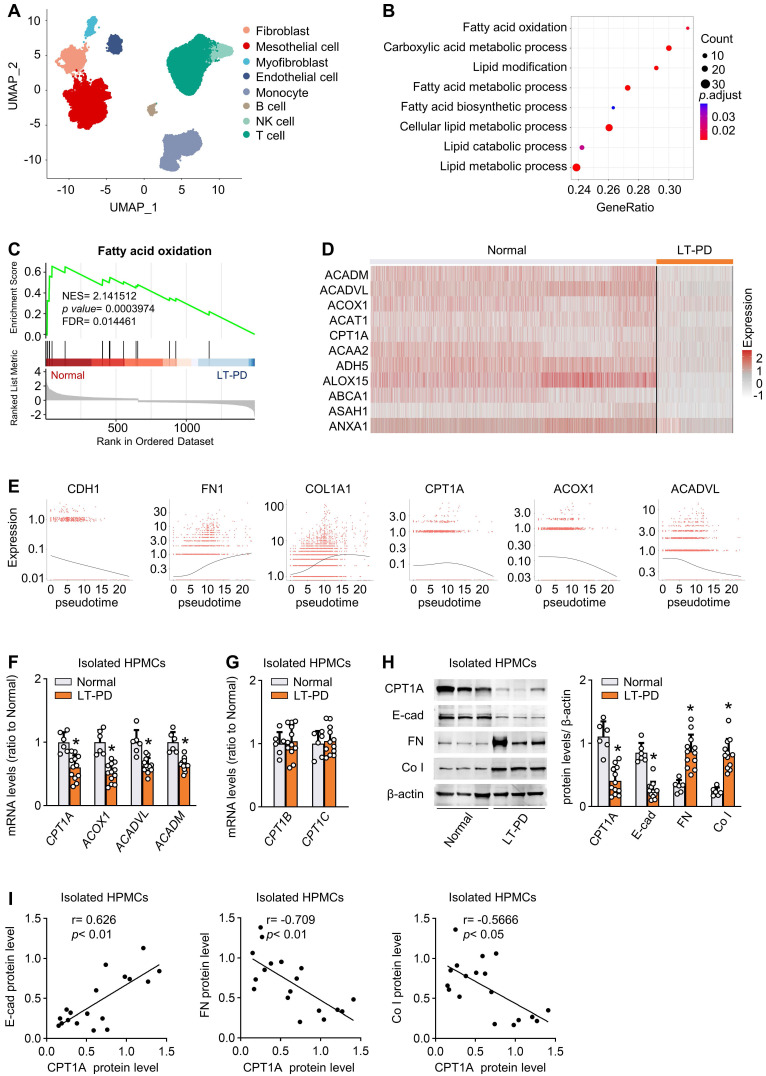
** Reduction of FAO in mesothelial cells characterizes patients on long-term PD**. (A) Uniform manifold approximation and projection (UMAP) shows overview of the cell clusters in the integrated single-cell transcriptomes derived from the effluent of patients on long-term PD (LT-PD, n = 4) and the normal peritoneum (normal, n = 3) using scRNA-seq data GSE130888. (B) Gene ontology analysis of mesothelial cells from patients on LT-PD and the normal peritoneum. The graph shows adjusted *P* values for the enrichment of a specific pathway. (C) GSEA plots demonstrating enrichment score (ES) of gene sets in the scRNA-seq data of mesothelial cells. Genes in each gene set are ranked by signal-to-noise ratio according to their differential expression between normal mesothelial cells and the long-term PD group. FDR: false discovery rate. (D) Heat map showing the expression of key FAO enzymes in mesothelial cells of normal and LT-PD groups. (E) Ordering of scRNA-seq expression data of peritoneal mesothelial cells according to the pseudotime produced by Monocle 3 package. Expression of epithelial marker, fibrotic marker, and FAO-related genes demonstrated along the trajectory. (F) Transcript levels of key FAO enzymes in human peritoneal mesothelial cells (HPMCs) isolated from normal and LT-PD group. Unpaired *t* test, *P < 0.05 *versus* normal group. (G) Transcript levels of CPT1B and CPT1C in isolated HPMCs. Unpaired *t* test. *P < 0.05 *versus* normal group. (H) Protein levels of CPT1A, E-cadherin (E-cad), fibronectin (FN) and collagen I (Co I) in isolated HPMCs. Unpaired *t* test, *P < 0.05 *versus* normal group. (I) Correlation analyses between transcript expression of CPT1A and fibrogenic markers in HPMCs. Spearman's correlation. Data expressed as mean ± SD in F-H (n = 6 in normal group and n = 12 in LT-PD group).

To investigate the FAO alterations of human mesothelial cells in response to long-term PD, we reanalyzed and annotated 41736 single cells from an elegant study 6, which includes 17108 cells from normal peritoneum (n = 3) and 24628 cells from effluents of patients undergoing long-term PD (PD ≥ 6 years, n = 4). Eight cell clusters were derived, covering diverse cell types in the peritoneum (Figure 1A and Figure S1A).

We focused on the metabolic genes detectable in mesothelial cells. In addition to glycolysis 6, gene ontology analysis highlighted specific changes in fatty acid metabolism and β-oxidation (Figure 1B). Gene set enrichment analysis confirmed the strong enrichment for FAO among the differently expressed pathways (Figure 1C). Further investigation revealed that levels of key FAO genes, including CPT1A, were markedly lower in the mesothelial population from the long-term PD group compared with the normal group (Figure 1D). In the other seven cell clusters, key FAO genes were not significantly downregulated (Figure S1B-H). Thus, long-term PD treatment could reduce FAO in mesothelial cells.

We next investigated whether this reduction in FAO is associated with the development of profibrotic phenotype in mesothelial cells. In long-term PD group, more than 79% of COL1A1+ cells were located in the mesothelial cell population and 19% of COL1A1+ cells are located in the fibroblast population (Figure S2A). These collagen-producing mesothelial cells also expressed VIM and FN1 (Figure S2B), supporting that effluent-derived mesothelial cells develop into a profibrotic phenotype in response to long-term PD and act as an important source of extracellular matrix contributing to fibrosis. We next performed cell trajectory analysis in mesothelial cells from both normal and long-term PD groups. As expected, mesothelial cells underwent transitions to a profibrotic phenotype (Figure S2D), with decreased expression of epithelial marker CDH1 (E-cadherin) (Figure 1E) and increased expression of profibrogenic transcription factors (SNAI1, SNAI2, ZEB2) and fibrogenic markers (FN1 and COL1A1) (Figure S2C and Figure 1E). Importantly, there is a negative correlation between trajectories of the fibrotic markers and the key FAO genes (Figure 1E).

Furthermore, by using flow cytometry, we isolated mesothelial cells from normal peritoneum (n = 6) and from effluents of patients on long-term PD (n = 12) (Figure S3A). Transcript levels of key FAO genes were reduced in mesothelial cells isolated from long-term PD group (Figure 1F). Notably, the mRNA level of CPT1A, the most abundant CPT1 isoform in mesothelial cells, was markedly downregulated in long-term PD group (Figure 1F and Figure S3B), whereas the mRNA level of CPT1B and CPT1C remained unchanged (Figure 1G). Changes in the protein expression of CPT1A paralleled changes in the mRNA expression (Figure 1H). This downregulation of key FAO genes in diseased mesothelial cells was accompanied by decreased expression of epithelial marker E-cadherin and increased expression of profibrogenic transcription factors and fibrogenic markers (Figure 1H-I; Figure S3C). Together, the development of the profibrotic phenotype in mesothelial cells is associated with a reduction in FAO, supporting our notion that a lower FAO in mesothelial cells contributes to the peritoneal fibrogenesis.

### Reduction of FAO in mesothelial cells characterizes mice with peritoneal fibrosis

**Figure 2:**
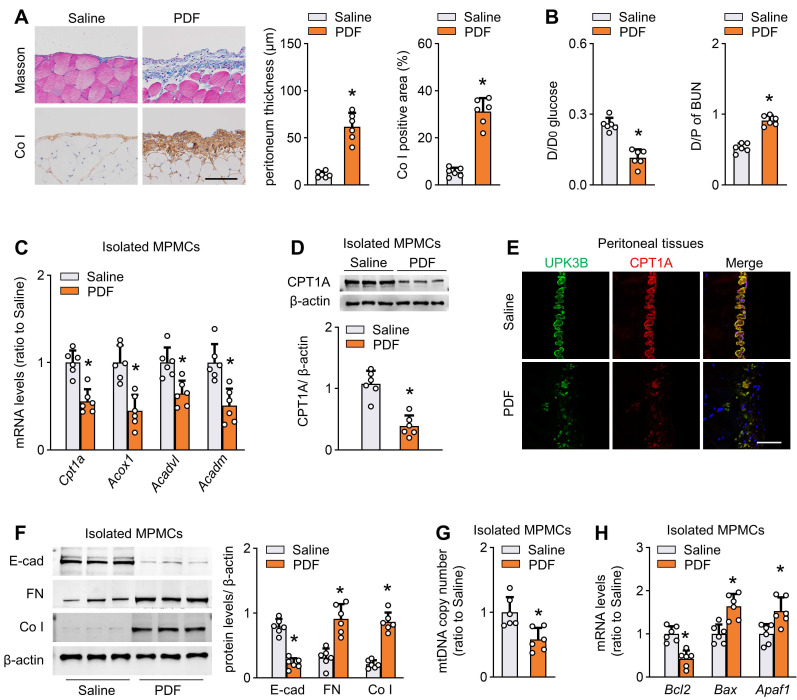
** Reduction of FAO in mesothelial cells characterizes mice with peritoneal fibrosis.** The mouse PD model of peritoneal fibrosis was induced by daily intraperitoneal injection of 4.25% PD fluid (PDF) for 6 weeks. Sham-operated mice were subject to daily intraperitoneal injection of saline for 6 weeks. (A) Peritoneal fibrosis, presented by Masson's trichrome staining and Co I staining: representative images and quantitative data. Scale bar, 100 μm. Unpaired *t* test. *P < 0.05 *versus* saline group. (B) Peritoneal permeability of glucose (D/D_0_) and blood urea nitrogen (BUN) (D/P) examined by modified peritoneal equilibration test. Unpaired *t* test. *P < 0.05 *versus* saline group. (C) Transcript levels of key FAO enzymes in isolated mouse peritoneal mesothelial cells (MPMCs). Unpaired *t* test. *P < 0.05 *versus* saline group. (D) CPT1A protein levels in isolated MPMCs. Unpaired *t* test. *P < 0.05 *versus* saline group. (E) Immunofluorescence staining for uroplakin 3B (UPK3B) and CPT1A in peritoneal tissues. (F) Protein levels of E-cad, FN and Co I in isolated MPMCs. Unpaired *t* test. *P < 0.05 *versus* saline group. (G) mtDNA copy number in isolated MPMCs. Unpaired *t* test. *P < 0.05 *versus* saline group. (H) Transcript levels of apoptosis markers in isolated MPMCs. Unpaired *t* test. *P < 0.05 *versus* saline group. Data expressed as mean ± SD in A-D, F-H (n = 6 in each group).

We established a mouse model of peritoneal fibrosis by intraperitoneally injecting mice with PD fluid containing 4.25% glucose daily for 6 weeks (PD mice). PD fluid treatment caused fibrotic lesions in the peritoneum, featured by significant thickening of parietal peritoneal tissue and increased submesothelial extracellular matrix deposition (Figure 2A and Figure S3D). Consistent with the histological data, peritoneal function was impaired in these PD mice, as indicated by decreased ultrafiltration rate and increased peritoneal permeability of glucose and blood urea nitrogen (Figure 2B and Figure S3E).

We next investigated whether FAO is altered in isolated mesothelial cells from the fibrotic PD mice. Transcript levels of key rate-limiting enzymes of FAO, including cpt1a, were markedly reduced in mesothelial cells from PD mice (Figure 2C). Importantly, CPT1A expression at the protein level paralleled changes at the mRNA level (Figure 2D). Whole-mount staining of peritoneal sections for UPK3B (marker of mesothelial cells) and CPT1A revealed downregulated immunolabeling of CPT1A in PD mice (Figure 2E and Figure S3F). The CPT1A activity was also reduced in mesothelial cells from PD mice (Figure S3G). Similarly, the reduction in key FAO gene levels was associated with downregulation of E-cadherin and upregulation of the profibrotic transcription factors and fibrogenic markers in mesothelial cells (Figure S3H and Figure 2F). Furthermore, mesothelial cells from PD mice showed a reduction in mitochondrial DNA copy number (Figure 2G) and expression of several key genes associated with mitochondrial biogenesis (Figure S3I), accompanied by upregulation of pro-apoptotic genes and downregulation of the anti-apoptotic gene Bcl2 (Figure 2H). Thus, all these data suggest that PD fluid suppresses FAO in mesothelial cells, which is associated with the development of peritoneal fibrosis.

### TGF-β1 decreases FAO and induces profibrotic phenotype in mesothelial cells

**Figure 3:**
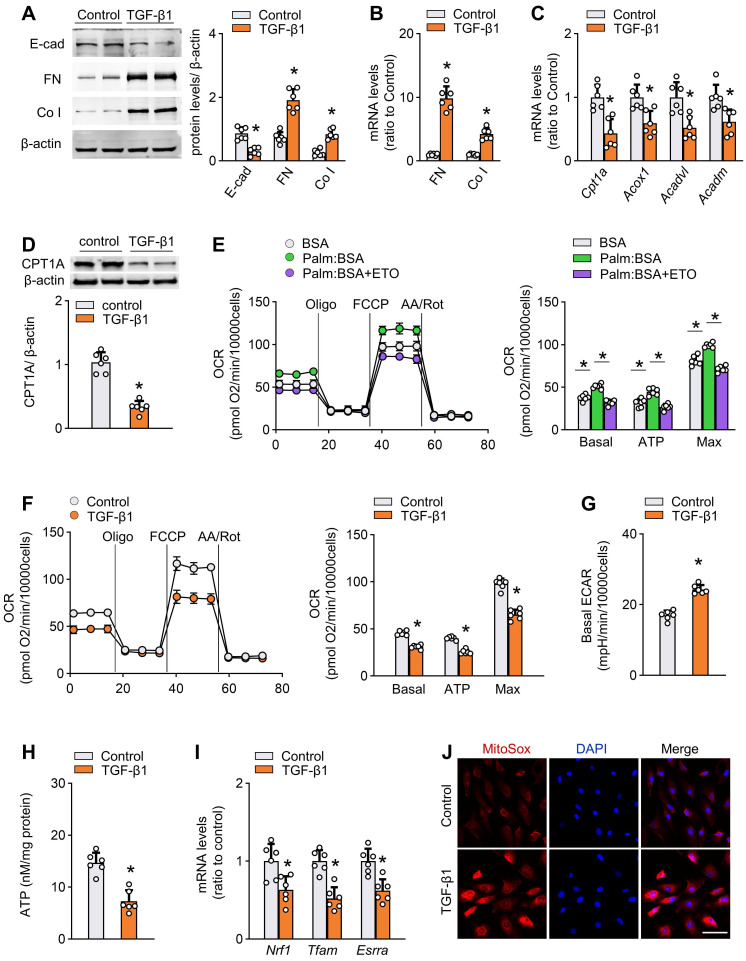
** TGF-β1 decreases FAO and promotes profibrotic phenotype in primary peritoneal mesothelial cells.** (A) Protein levels of E-cad, FN and Co I in primary MPMCs treated with or without TGF-β1. Unpaired *t* test. *P < 0.05 *versus* control (PBS). (B) Transcript levels of FN and Co I in primary MPMCs treated with or without TGF-β1. Unpaired *t* test. *P < 0.05 *versus* control. (C) Transcript levels of key FAO enzymes in primary MPMCs treated with or without TGF-β1. Unpaired *t* test. *P < 0.05 *versus* control. (D) CPT1A protein level in primary MPMCs treated with or without TGF-β1. Unpaired *t* test. *P < 0.05 *versus* control. (E) Oxygen consumption rate (OCR) measurement of Mito Stress assay in primary MPMCs. Where indicated, cells are pretreated with palmitate-BSA FAO substrate (Palm:BSA, 30 μL) or the CPT1 inhibitor etomoxir (Eto, 4 μM). One-way ANOVA with Bonferroni correction. *P < 0.05. (F) OCR measurement in primary MPMCs treated with or without TGF-β1. Unpaired *t* test. *P < 0.05 *versus* control. (G) Quantification of ECAR measurement in F. Unpaired *t* test. *P < 0.05 *versus* control. (H) ATP levels in primary MPMCs treated with or without TGF-β1. Unpaired *t* test. *P < 0.05 *versus* control. (I) Transcript levels of mitochondrial biogenesis-associated genes in primary MPMCs treated with or without TGF-β1. Unpaired *t* test. *P < 0.05 *versus* control. (J) Representative images of MitoSOX staining in primary MPMCs treated with or without TGF-β1. Scale bar, 50 μm. Data expressed as mean ± SD in A-I (n = 6 in each group).

Activation of TGF-β1 is an important early event that mediates peritoneal fibrogenesis during PD 9. We confirmed that TGF-β1 levels were increased in both peritoneal tissue and effluent of PD mice (Figure S3J). Therefore, to further investigate the FAO function during peritoneal fibrogenesis and to isolate it from systemic influence, we evaluated the effect of TGF-β1 *in vitro* on two mesothelial cell subtypes: primary mouse mesothelial cells isolated from peritoneum (Figure S4A), and a human mesothelial cell line Met-5A cells. Treatment of both mouse mesothelial cells and Met-5A cells with TGF-β1 (5 ng/mL for 24 h) induced profibrotic phenotypic changes, featured by loss of an epithelial phenotype and upregulation of profibrotic transcription factors and fibrotic markers (Figure 3A-B, Figure S4B, Figure S5A). The induction of fibrotic markers in response to TGF-β1 was dose-dependent (0-10 ng/mL; Figure S4C).

More importantly, in each of the cells, treatment with TGF-β1 (5 ng/mL) for 24 h resulted in a marked decrease in the mRNA expression of key FAO pathway enzymes, including CPT1A, ACOX1, ACADVL, and ACADM (Figure 3C, Figure S5B). Immunoblot studies confirmed lower protein levels of CPT1A in TGF-β1-treated cells (Figure 3D and Figure S5C). The expression of CPT1A in response to TGF-β1 was dose-dependent (0-10 ng/mL; Figure S4D).

To analyze the metabolism quantitatively, we determined the oxygen consumption rate (OCR; a representation of mitochondrial activity) and extracellular acidification rate (ECAR; a surrogate for glycolytic rate) in both primary mouse mesothelial cells and Met-5A cells. As shown in Figure 3E and Figure S5D, the basal and maximum OCR were markedly increased when palmitate was added to the cells, indicating that mesothelial cells metabolized palmitate efficiently. This increase in OCR was sensitive to the CPT1 inhibitor etomoxir, confirming its specificity. Strikingly, cells treated with TGF-β1 had a lower basal and maximum OCR (Figure 3F and Figure S5E) but a higher ECAR (Figure 3G and Figure S5F), suggesting low activity of FAO. These data support our hypothesis that TGF-β1 lowers FAO in mesothelial cells.

Consistent with the FAO depression, there were reduced ATP content (Figure 3H), downregulation of mitochondrial biogenesis-associated genes (Figure 3I), enhanced generation of mitochondrial superoxide (Figure 3J), decreased mitochondrial DNA copy number (Figure S4E), reduced expression of anti-apoptotic gene, and increased expression of pro-apoptotic genes (Figure S4F) in TGF-β1-treated primary mouse mesothelial cells.

**Figure 4:**
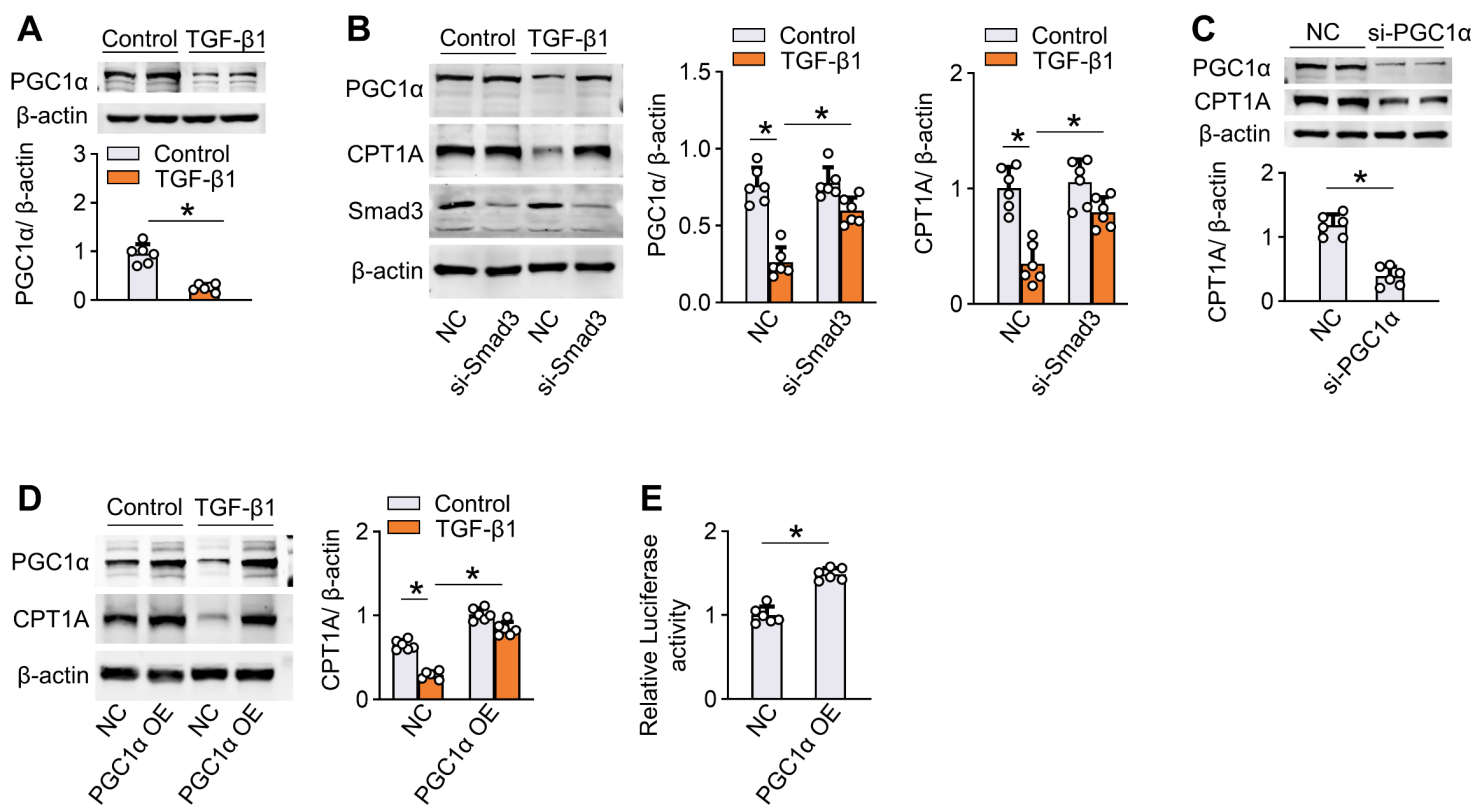
** TGF-β1 downregulates CPT1A via a SMAD3/PGC-1α pathway in mesothelial cells.** (A) Incubation of Met-5A cells with TGF-β1 for 24 h downregulates the protein level of PGC1α. Unpaired *t* test, *P < 0.05. (B) Knockdown of Smad3 in Met-5A cells is achieved by transfecting cells with siRNA targeted to Smad3 (si-Smad3). Transfection of Met-5A cells with si-Smad3 (*versus* negative control, NC) reverses the ability of TGF-β1 to reduce the expression of PGC-1α and CPT1A. One-way ANOVA with Bonferroni correction, *P < 0.05. (C) Knockdown of PGC-1α by si-PGC1α in Met-5A cells downregulates the expression of CPT1A. (D) Overexpression of PGC-1α in Met-5A cells is achieved by transfecting cells with pcDNA3.1-PGC1α plasmids (PGC-1α OE). Transfection of Met-5A cells with PGC-1α OE (*versus* pcDNA3.1 empty vector, NC) reverses the ability of TGF-β1 to reduce the expression of CPT1A. One-way ANOVA with Bonferroni correction, *P < 0.05. (E) Luciferase activity in Met-5A cells transfected with PGC-1α OE. Unpaired *t* test, *P < 0.05. Data expressed as mean ± SD in A-E (n = 6 in each group).

We also analyzed the molecular mechanism underlying TGF-β1-induced suppression of FAO in mesothelial cells. Incubation of human Met-5A cells with TGF-β1 (5 ng/mL) for 24 h decreased the protein level of PPARγ coactivator-1α (PGC1α) (Figure 4A) that acts as a transcriptional factor regulating gene expression of CPT1A 12,19,20. Knockdown of Smad3, a transcriptional regulator of PGC1α 12,21,22, in mesothelial cells reversed the ability of TGF-β1 to reduce the expression of PGC-1α and CPT1A (Figure 4B). Knockdown of PGC-1α in mesothelial cells decreased the CPT1A expression (Figure 4C), whereas overexpression of PGC-1α rescued the TGF-β1-induced downregulation of CPT1A (Figure 4D). Luciferase reporter assays further showed that overexpression of PGC-1α enhanced CPT1A promoter activity in mesothelial cells (Figure 4E). Thus, the reduction of FAO by the profibrotic inducer TGF-β1 is in large part mediated *via* a SMAD3/PGC-1α pathway. Altogether, the ability of TGF-β1 to decrease FAO in mesothelial cells highlights the potential role of defective FAO in mesothelial cells in the development of peritoneal fibrosis.

### Restoration of CPT1A expression reverses TGF-β1-induced FAO suppression and profibrotic phenotype in mesothelial cells

**Figure 5:**
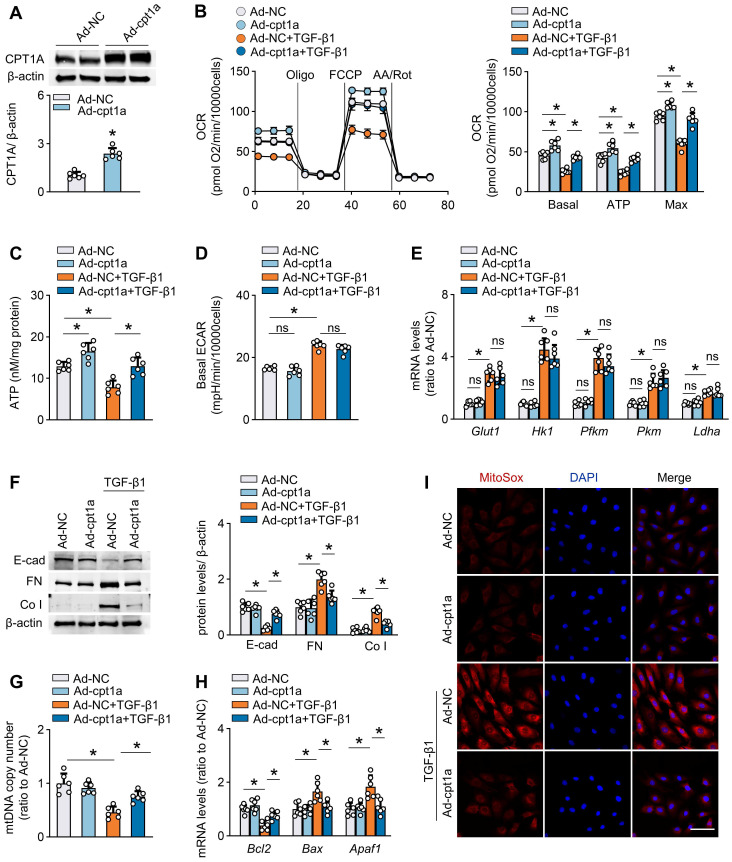
** Restoration of CPT1A expression reverses TGF-β1-induced FAO suppression and profibrotic phenotype in primary peritoneal mesothelial cells.** Primary MPMCs are transfected with either adenovirus carrying CPT1A (Ad-CPT1A) or negative control (Ad-NC). (A) Immunoblots showing upregulation of CPT1A in MPMCs transfected with Ad-CPT1A. Unpaired *t* test, *P < 0.05. (B and C) Transfection of MPMCs with Ad-CPT1A (*versus* Ad-NC) increases the FAO-associated OCR (B) and elevates ATP levels (C). One-way ANOVA with Bonferroni correction. *P < 0.05. (D and E) Overexpression of CPT1A in MPMCs does not alter ECAR (D) and glycolysis-related genes expression (E). One-way ANOVA with Bonferroni correction in D and Kruskal-Wallis test in E. *P < 0.05. ns, no significance. (F-I) MPMCs overexpressing CPT1A are protected from TGF-β1-induced detrimental changes, including induction of profibrotic marker expression (F), mitochondrial DNA copy number (G), level of apoptosis-associated genes (H), and mitochondrial superoxide generation (I; Scale bar, 50 μm). One-way ANOVA with Bonferroni correction, *P < 0.05. Data expressed as mean ± SD in A-D, F-H, and median with IQR in E (n = 6 in each group).

To investigate whether a defective FAO is involved in the TGF-β1-induced profibrotic phenotype in mesothelial cells, we established mouse mesothelial cells overexpressing CPT1A by transfecting these cells with an adenovirus carrying CPT1A (Ad-CPT1A). As demonstrated, transfection of mesothelial cells with Ad-CPT1A (*versus* adenovirus control) upregulated CPT1A expression (Figure 5A and Figure S6A), increased the FAO-associated OCR (Figure 5B), and elevated ATP levels (Figure 5C), suggesting a key role of CPT1A in regulating mesothelial FAO. Furthermore, overexpression of CPT1A in mouse mesothelial cells reversed TGF-β1 (5 ng/mL)-induced decrease in OCR, and restored ATP levels (Figure 5B-C). However, ECAR and the expression of glycolysis-related genes were unaltered in mesothelial cells overexpressing CPT1A (Figure 5D-E). Thus, rescue of CPT1A expression specifically improves FAO in mesothelial cells treated with TGF-β1.

Furthermore, mouse mesothelial cells overexpressing CPT1A were protected from TGF-β1 (5 ng/mL)-induced detrimental changes, including the expression of profibrogenic transcription factors and fibrotic markers (Figure S6B, Figure 5F), NADPH production (Figure S6C), mitochondrial DNA copy number (Figure 5G), mitochondrial superoxide generation (Figure 5I), and levels of mitochondrial biogenesis-associated genes and apoptosis-associated markers (Figure S6D-E, Figure 5H). We also examined the effect of CPT1A overexpression in Met-5A cells. Genetic overexpression of CPT1A reversed the TGF-β1 (5 ng/mL)-induced decrease in OCR (Figure S6F-G) and reduced the expression of fibrotic markers (Figure S6H) in these cells. Thus, restoration of CPT1A-mediated FAO reverses TGF-β1-induced profibrotic phenotype in mesothelial cells.

We then investigated how FAO regulates the profibrotic phenotype in mesothelial cells. The FAO occurs mainly in the mitochondria and functions as essential source of the antioxidant NADPH, linking FAO to oxidative stress regulation 23,24. Importantly, the transcription factor Snail, Slug, ZEB2 are recently found to be redox-sensitive 25, enabling the elucidation of the molecular basis underlying the CPT1A-modulated profibrotic process from a redox perspective. Indeed, the antioxidant Mito-TEMPO (100 μM), which eliminates mitochondrial ROS levels (Figure S7A), reverses the TGF-β1 (5 ng/mL)-induced upregulation of profibrogenic transcription factors and its ability to promote mesothelial transition toward the profibrotic phenotype (Figure S7B-C). Notably, CPT1A expression abolished the protective responses induced by Mito-TEMPO in TGF-β1-treated mesothelial cells (Figure S7D). Thus, CPT1A regulates the profibrotic phenotype of mesothelial cells through a ROS-dependent pathway under PD conditions. Taken together, a defective FAO is involved in TGF-β1-induced profibrotic phenotype in mesothelial cells, supporting that repression of CPT1A-driven FAO in mesothelial cells could be a critical factor contributing to the profibrotic process in the peritoneum.

### Restoration of CPT1A expression improves FAO and protects against fibrosis in mouse peritoneum

We next tested whether rescue of CPT1A expression *in vivo* protects peritoneum from the development of fibrosis. We used both transgenic and pharmacological approaches to investigate this issue.

**Figure 6:**
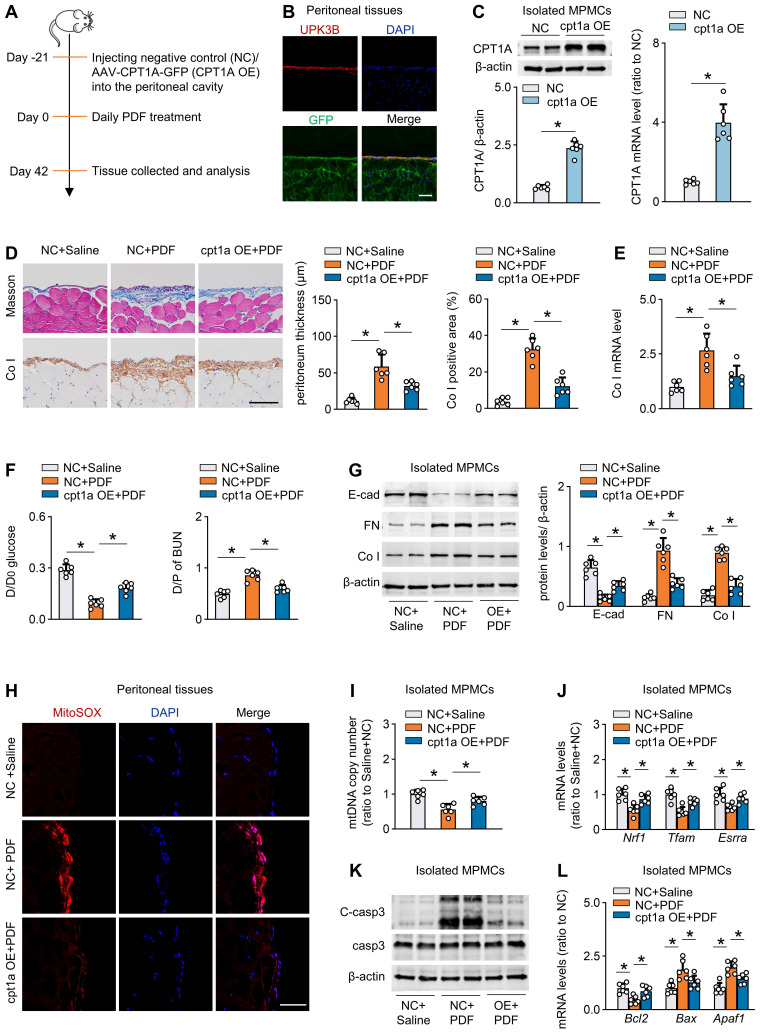
** Restoration of CPT1A expression improves FAO and protects against fibrosis in mouse peritoneum**. (A) Outline of experimental protocol: Restoration of CPT1A in mesothelial cells in PD mice is achieved by injecting AAV-CPT1A-GFP (CPT1A OE) into peritoneal cavity of mice 21 days before daily PDF treatment. (B) Representative images show immunostaining of UPK3B and GFP in peritoneum of mouse treated with AAV-GFP. Scale bar, 100μm. (C) CPT1A protein level and transcript level in isolated MPMCs. Unpaired *t* test, *P < 0.05. (D) Peritoneal fibrosis, presented by Masson's trichrome staining and Co I staining in mice: representative images and quantitative data. Scale bar, 100 μm. One-way ANOVA with Bonferroni correction. *P < 0.05. (E) Transcript level of Co I in peritoneal tissues. One-way ANOVA with Bonferroni correction. *P < 0.05. (F) Peritoneal permeability of glucose (D/D0) and BUN (D/P) examined by modified peritoneal equilibration test. One-way ANOVA with Bonferroni correction. *P < 0.05. (G) Protein levels of E-cad, FN and Co I in isolated MPMCs. One-way ANOVA with Bonferroni correction. *P < 0.05. (H) Representative images show MitoSOX staining in mouse peritoneal tissues. Scale bar, 50 μm. (I) mtDNA copy number in isolated mouse peritoneal mesothelial cells. One-way ANOVA with Bonferroni correction. *P < 0.05. (J) Transcript levels of mitochondrial biogenesis-associated genes in isolated MPMCs. One-way ANOVA with Bonferroni correction. *P < 0.05. (K) Protein levels of cleaved capase3 (C-casp3) and casp3 in isolated MPMCs. (L) Transcript levels of apoptosis markers in isolated MPMCs. One-way ANOVA with Bonferroni correction. *P < 0.05. Data expressed as mean ± SD in C-G and I-L (n = 6 in each group).

First, to efficiently overexpress CPT1A *in vivo*, we used an adeno-associated virus 9 (AAV9)-mediated therapy (Figure 6A). We validated the transfection efficiency of AAV9 in mouse peritoneal mesothelial cells by intraperitoneal injection of AAV9-GFP. Three weeks after injection of the virus, whole-mount staining of peritoneal sections for UPK3B (marker of mesothelial cells) and GFP revealed that mesothelial cells were the major cells expressing GFP (Figure 6B). We then generated AAV9-CPT1A, and showed a pronounced upregulation of CPT1A in isolated mouse peritoneal mesothelial cells after single-dose intraperitoneal injection of AAV9-CPT1A (Figure 6C). PD fluid caused significant peritoneal fibrosis and impaired peritoneal function. However, these PD fluid-induced effects were largely improved in mice transfected with AAV9-CPT1A (Figure 6D-F, Figure S8A-B). This transgenic expression of CPT1A in PD fluid-treated mice also reduced the expression of profibrogenic transcription factors (Figure S8C) and fibrotic markers (Figure 6G), improved mitochondrial function (Figure 6H-J), downregulated pro-apoptotic gene levels and restored the anti-apoptotic gene expression (Figure 6K-L, Figure S8D). In contrast, CPT1A overexpression unaltered the enzyme levels related to glycolysis pathway in mesothelial cells (Figure S8E). Thus, transgenic overexpression of CPT1A in mesothelial cells, which does not impinge on glycolysis, protects mice from the development of PD fluid-induced peritoneal fibrosis.

**Figure 7:**
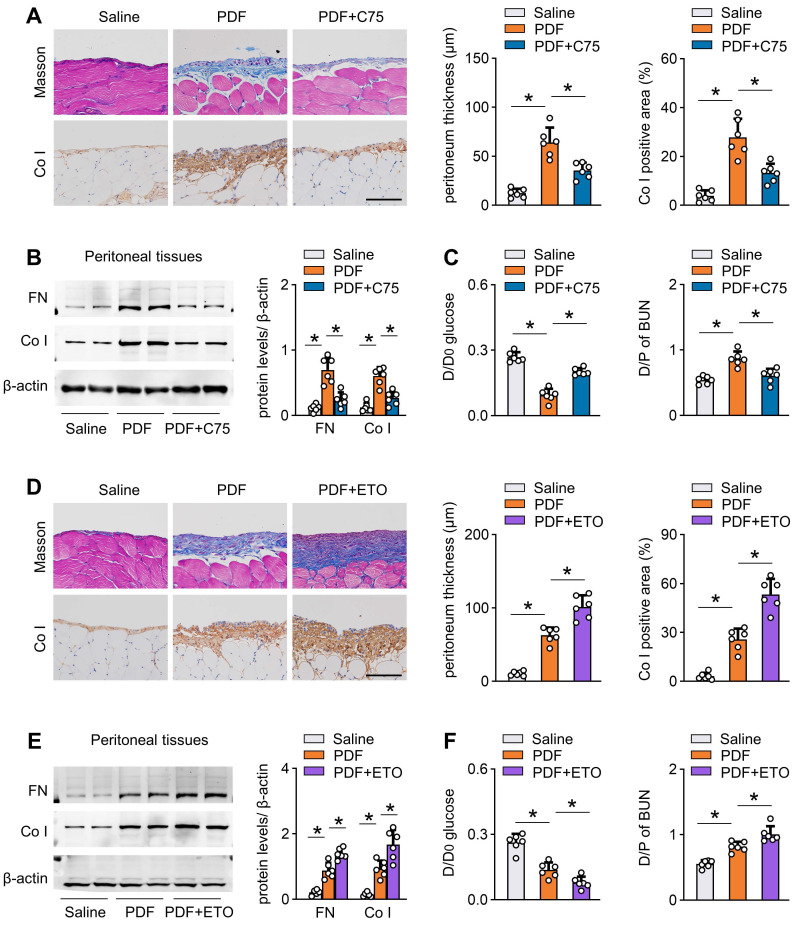
** Pharmacological improvement of FAO protects against fibrosis in mouse peritoneum.** (A-C) Pharmacological improvement of FAO is achieved by intraperitoneal injection of mice with C75 three times per week starting 1 day after PDF treatment. Peritoneal fibrosis, presented by Masson's trichrome staining and Co I staining in mice: representative images and quantitative data; Scale bar, 100 μm (A). Protein levels of FN and Co I in mouse peritoneal tissues (B). Peritoneal permeability of glucose (D/D0) and BUN (D/P) examined by modified peritoneal equilibration test (C). One-way ANOVA with Bonferroni correction. *P < 0.05. (D-F) Pharmacological inhibition of FAO is achieved by intraperitoneally injecting mice with etomoxir three times per week starting 1 day after PDF treatment. Peritoneal fibrosis, presented by Masson's trichrome staining and Co I staining in mice: representative images and quantitative data; Scale bar, 100 μm (D). Protein levels of FN and Co I in mouse peritoneal tissues (E). Peritoneal permeability of glucose (D/D0) and BUN (D/P) examined by modified peritoneal equilibration test (F). One-way ANOVA with Bonferroni correction. *P < 0.05. Data expressed as mean ± SD in A-F (n = 6 in each group).

Next, we investigated whether pharmacological activation of CPT1A can protect mice from development of peritoneal fibrosis. We intraperitoneally injected mice with C75 three times per week starting 1 day after PD fluid treatment. C75 treatment reversed the PD fluid-induced reduction in CPT1A activity (Figure S8G). C75 treatment also improved peritoneal fibrosis and function in this PD model (Figure 7A-C, Figure S8H-I). Similarly, C75 administration of PD fluid-treated mice did not affect the key glycolytic enzyme expression (Figure S8J).

To provide further evidence that peritoneal fibrosis is linked to a defect in FAO, we tested the effect of etomoxir, a specific inhibitor of CPT1. Mice injected with etomoxir developed more severe peritoneal injury after PD fluid treatment, featured by markedly higher peritoneal expression of extracellular matrix and worse peritoneal function (Figure 7D-F, Figure S8K-L). Together, our results concur in demonstrating that inhibition of CPT1A in PD fluid-treated mice dysregulates FAO and thereby contributes to peritoneal fibrosis in this mouse model of PD.

## Discussion

**Figure 8:**
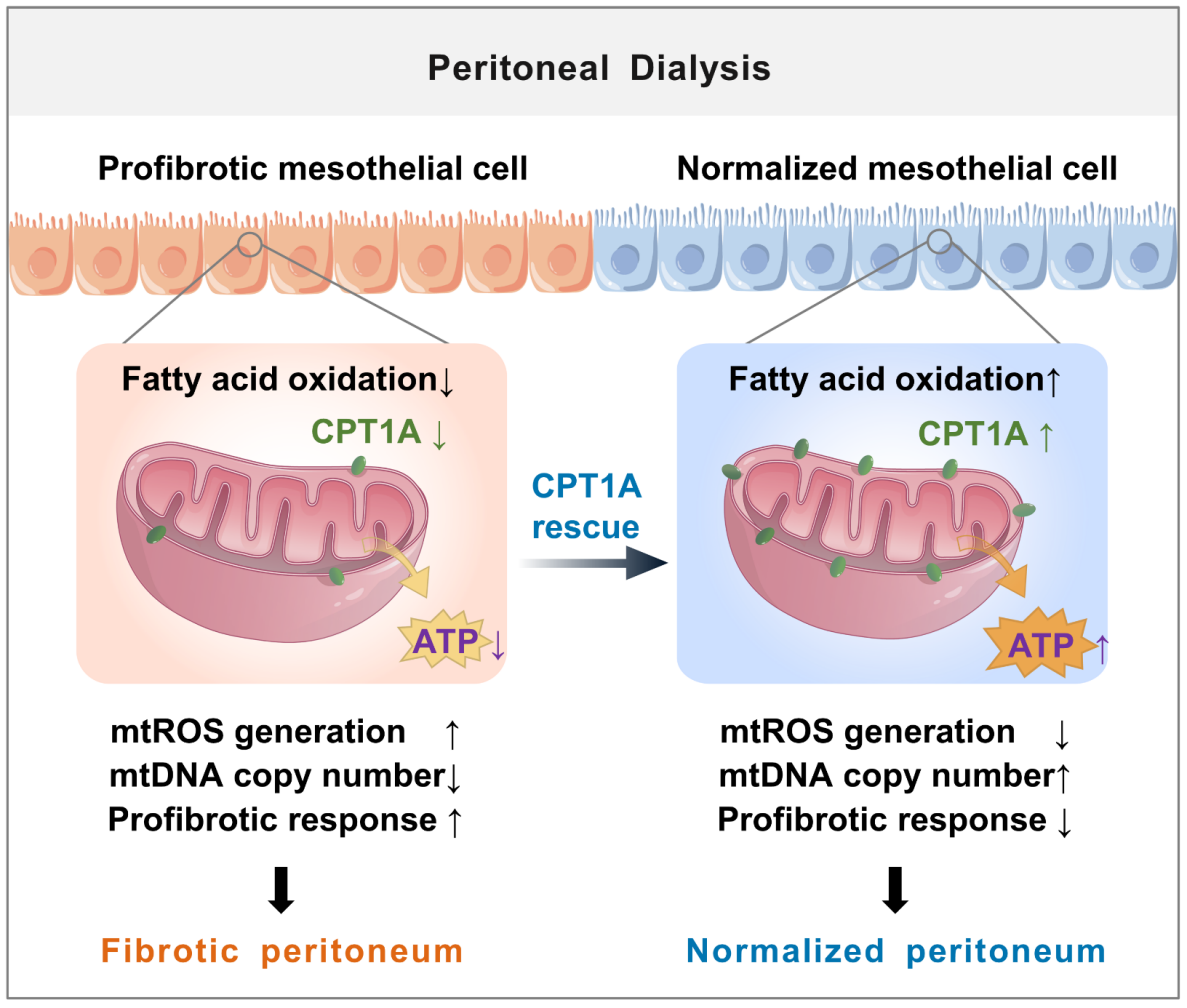
** Schematic diagram summarizing a novel role for disrupted FAO of mesothelial cells in the development of peritoneal fibrosis during long-term PD**. We demonstrate a defective FAO in mesothelial cells from both patients on long-term PD and from experimental PD mice with peritoneal fibrosis. The reduction in FAO induces a profibrotic phenotype in these cells, which contributes to the mitochondrial dysfunction and development of peritoneal fibrosis. Improving FAO by restoration of CPT1A expression in mesothelial cells impairs their transition toward profibrotic phenotype, thereby lessening the peritoneal fibrosis in the experimental PD mice.

The major new finding from our study is of a novel role for disrupted FAO of mesothelial cells in the development of peritoneal fibrosis (illustrated in Figure 8). We demonstrate a defective FAO in mesothelial cells from both patients on long-term PD and from experimental PD mice with peritoneal fibrosis. The reduction in FAO induces a profibrotic phenotype in these cells, which contributes to the development of peritoneal fibrosis. Restoration of FAO by overexpressing CPT1A or pharmacologically activating CPT1A in mesothelial cells, impairs their transition toward profibrotic phenotype, thereby lessening the peritoneal fibrosis in the experimental PD mice. Our data demonstrate for the first time that disruption of CPT1A-driven FAO in mesothelial cells might be a critical mechanism underlying PD-associated peritoneal fibrosis.

Here, we study changes of the metabolic pathway in human mesothelial cells in PD patients by analyzing a scRNA-seq data from an elegant study 6. Our unbiased analysis indicates a strong correlation between a defective FAO and a profibrotic phenotype in mesothelial cells. The importance of mesothelial profibrotic phenotype for development of the peritoneal fibrosis has long been recognized 2,6,7. It is therefore reasonable to postulate that this lower FAO in mesothelial cells acts as crucial participant in the fibrotic fate of the peritoneum. In support of this notion, both primary mouse mesothelial cells and human MeT-5A cells are able to use fatty acid as an energy substrate. Overexpression of CPT1A in mesothelial cells, which prevents the TGF-β1-induced decrease in FAO activity, restores cellular ATP levels, and reverses the TGF-β1-induced transition toward the profibrotic phenotype. The functional importance of FAO in PD is further shown by improvement of the FAO in mesothelial cells, either by genetic or pharmacological restoration of CPT1A, which greatly attenuates the profibrotic effects and lessens the peritoneal fibrosis. In the setting of PD, the presence of a hyperglycolytic state in mesothelial cells has recently been described 6,26. However, activity of other metabolic pathways in PD has not been studied. In this study, we demonstrate the presence of a defective FAO in mesothelial cells in PD, which contributes to the development of peritoneal fibrosis.

More interestingly, although a reciprocal regulation of FAO and glycolysis, known as the Randle cycle 27, has been demonstrated in the muscle and adipose tissue, our data show that it does not occur in peritoneal mesothelial cells. We demonstrate that maneuvers to restore FAO in mesothelial cells do not significantly alter their glycolytic activity. These findings are similar to those in hepatic stellate cells, where overexpression of CPT1A stimulates OCR but does not alter ECAR 28. These results suggest that each cell type may have a specific metabolic rewiring in response to injury or treatment, and highlight that a defective FAO alone is sufficient to reprogram mesothelial cells into a profibrotic phenotype and thus plays a critical role in determining the fibrotic process during PD.

Mesothelial mitochondrial abnormalities are common features in the pathogenesis of peritoneal fibrosis 10,29,30. Cellular pathways that promote mesothelial cell injury can compromise mitochondrial homeostasis, contributing to the development of profibrotic phenotype in mesothelial cells and the deterioration of peritoneal function 31-33. The new finding is that restoring FAO in mesothelial cells can reduce mitochondrial abnormalities caused by PD. The FAO occurs mainly in the mitochondria and functions as essential source of ATP and the antioxidant NADPH 23,24. In the setting of PD, restoration of mesothelial FAO increases ATP production, providing energy for mitochondrial biogenesis 17,34,35. Furthermore, restoring FAO in mesothelial cells, which increases NADPH generation, reverses PD-induced mitochondrial superoxide production. The elevated ATP production and decreased mitochondrial oxidative stress contribute to increased mitochondrial biogenesis, reduced mitochondrial DNA damage, and downregulation of redox-sensitive transcription factors of mesothelial-to-mesenchymal transition- all processes that maintain mitochondrial homeostasis 25,36 and preserve peritoneal structure during PD.

An interesting finding is that TGF-β1 seems to be an important upstream modulator of FAO in peritoneal mesothelial cells. TGF-β1 is a well-established principal driver of fibrosis in many organs, including the peritoneum 37,38. TGF-β1 has also been shown to stimulate glycolysis in the peritoneum 6. Levels of TGF-β1 are consistently increased in effluent from patients on long-term PD 39 and in peritoneal tissues from experimental PD mice 40,41. Here, prolonged incubation of both primary mouse mesothelial cells and human MeT-5A cells with TGF-β1, downregulates the expression of key FAO enzymes (including CPT1A), impairs FAO and induces the profibrotic response in these cells. These TGF-β1-induced detrimental changes could be regulated by CPT1A, since rescue of CPT1A expression in mesothelial cells specifically improves FAO and prevents the profibrotic response. We further attribute the intracellular TGF-β1 signaling underlying the downregulation of CPT1A in mesothelial cells to the activation of SMAD3, which reduces the expression of PGC1α that is known to regulate CPT1A gene transcription 12,21,22. Interruption of this signaling pathway corrects the reduction in CPT1A of mesothelial cells with TGF-β1. Thus, enhanced TGF-β1 production during PD impairs CPT1A-driven FAO that promotes peritoneal fibrosis.

The nature of the peritoneal cells that produce extracellular matrix during peritoneal fibrosis is still under debate. Lineage tracing studies in mice suggest that peritoneal myofibroblasts derived from submesothelial fibroblasts are the major contributors to fibrosis 42. It has also been proposed that peritoneal mesothelial cells evolve into myofibroblast-like producers of extracellular matrix in response to PD treatment 7. Our analyses of scRNA-seq data, consistent with *Si et al, 2019* 6, support that PD effluent-derived mesothelial cells may act as an important source of extracellular matrix contributing to fibrosis due to their robust production of collagens. However, it is still possible that, in the setting of PD, mesothelial cells display a profibrotic secretome that could activate resident peritoneal fibroblasts and thereby induce their synthetic capacity of extracellular matrix. Indeed, mesothelial cells in PD effluent exhibit a specific secretome with increased expression of profibrogenic growth factors TGF-β1 and CTGF (Figure S9A), which are well-established principal drivers of fibrosis via promotion of fibroblast proliferation and collagen production 43. This mesothelial cell-mediated activation of fibroblasts is also regulated by FAO. Restoration of FAO in the TGF-β1-treated mesothelial cells modifies their secretome, contributing to reduced fibroblast activation and collagen production (Figure S9B-D). Together, all these observations identify the mesothelial cells as critical participants in the production of extracellular matrix and the development of peritoneal fibrosis.

Our studies could have several important clinical implications. First, enhancing FAO might be of therapeutic benefit for patients on PD. Specifically, the CPT1A could be a therapeutic target potentially achieved by genetic overexpression or pharmacological activation, to prevent the progressive peritoneal fibrosis in PD. Next, pharmacological blockade of FAO has gained attention for the its efficacy to treat heart diseases or cancer 44-48. The finding that PD mice injected with CPT1A inhibitor show enhanced peritoneal fibrosis, warrants future studies to determine the long-term consequences of FAO inhibitors in related patients, especially in PD patients.

This study also has limitations. The human scRNA-seq data cited in this manuscript includes cells from normal human peritoneum and from the effluent of PD patients. The PD effluent may consist mainly of cells shed from the peritoneum, which has a different composition to the cells in the peritoneum. Although our findings from the scRNA-seq data was corroborated in PD mice and in mesothelial cells incubated with TGFβ1, the differences between these shed cells collected in the effluent and peritoneal cells not captured in the effluent require further investigation. In addition, we do not measure FAO rates using radiolabeled palmitate in mesothelial cells from PD mice. However, the activity of CPT1A, a rate-limiting enzyme in FAO, has been measured. We demonstrate that CPT1A activity is reduced in mesothelial cells from PD mice and that this is reversed by C75 treatment.

In sum, our study demonstrates a disrupted mesothelial FAO in PD that causes metabolic reprogramming of mesothelial cells, leading to development of a profibrotic phenotype. Restoring FAO by genetic and pharmacological means reverses the profibrotic responses in mesothelial cells and protects the peritoneum from the development of fibrosis. Thus, maintaining the proper degree of FAO is a potential therapeutic goal to prevent peritoneal fibrosis in patients on PD.

## Supplementary Material

Supplementary methods, figures and tables.

Supplementary table 1.

## Abbreviations

FAO: fatty acid oxidation
CPT1A: Carnitine palmitoyltransferase 1A
TGF-β1: transforming growth factor β1
AAV: adeno-associated virus
OCR: oxygen consumption rate
ECAR: extracellular acidification rate
PD: peritoneal dialysis
scRNA-seq: single cell RNA-sequence
Ad-CPT1A: adenovirus carrying CPT1A

## References

[1] Cho Y, Bello AK, Levin A, Lunney M, Osman MA, Ye F (2021). Peritoneal Dialysis Use and Practice Patterns: An International Survey Study. Am J Kidney Dis.

[2] Teitelbaum I (2021). Peritoneal Dialysis. N Engl J Med.

[3] Cho Y, Johnson DW (2018). PD Solutions and Peritoneal Health. Clin J Am Soc Nephrol.

[4] Brimble KS, Walker M, Margetts PJ, Kundhal KK, Rabbat CG (2006). Meta-analysis: peritoneal membrane transport, mortality, and technique failure in peritoneal dialysis. J Am Soc Nephrol.

[5] Devuyst O, Margetts PJ, Topley N (2010). The pathophysiology of the peritoneal membrane. J Am Soc Nephrol.

[6] Si M, Wang Q, Li Y, Lin H, Luo D, Zhao W (2019). Inhibition of hyperglycolysis in mesothelial cells prevents peritoneal fibrosis. Sci Transl Med.

[7] Yanez-Mo M, Lara-Pezzi E, Selgas R, Ramirez-Huesca M, Dominguez-Jimenez C, Jimenez-Heffernan JA (2003). Peritoneal dialysis and epithelial-to-mesenchymal transition of mesothelial cells. N Engl J Med.

[8] Helmke A, Nordlohne J, Balzer MS, Dong L, Rong S, Hiss M (2019). CX3CL1-CX3CR1 interaction mediates macrophage-mesothelial cross talk and promotes peritoneal fibrosis. Kidney Int.

[9] Zhou Q, Bajo MA, Del PG, Yu X, Selgas R (2016). Preventing peritoneal membrane fibrosis in peritoneal dialysis patients. Kidney Int.

[10] Ramil-Gomez O, Rodriguez-Carmona A, Fernandez-Rodriguez JA, Perez-Fontan M, Ferreiro-Hermida T, Lopez-Pardo M (2021). Mitochondrial Dysfunction Plays a Relevant Role in Pathophysiology of Peritoneal Membrane Damage Induced by Peritoneal Dialysis. Antioxidants (Basel).

[11] Busnadiego O, Loureiro-Alvarez J, Sandoval P, Lagares D, Dotor J, Perez-Lozano ML (2015). A pathogenetic role for endothelin-1 in peritoneal dialysis-associated fibrosis. J Am Soc Nephrol.

[12] Kang HM, Ahn SH, Choi P, Ko YA, Han SH, Chinga F (2015). Defective fatty acid oxidation in renal tubular epithelial cells has a key role in kidney fibrosis development. Nat Med.

[13] Lopaschuk GD, Karwi QG, Tian R, Wende AR, Abel ED (2021). Cardiac Energy Metabolism in Heart Failure. Circ Res.

[14] Cortassa S, Sollott SJ, Aon MA (2017). Mitochondrial respiration and ROS emission during beta-oxidation in the heart: An experimental-computational study. Plos Comput Biol.

[15] Pike LS, Smift AL, Croteau NJ, Ferrick DA, Wu M (2011). Inhibition of fatty acid oxidation by etomoxir impairs NADPH production and increases reactive oxygen species resulting in ATP depletion and cell death in human glioblastoma cells. Biochim Biophys Acta.

[16] Schlaepfer IR, Joshi M (2020). CPT1A-mediated Fat Oxidation, Mechanisms, and Therapeutic Potential. Endocrinology.

[17] Miguel V, Tituana J, Herrero JI, Herrero L, Serra D, Cuevas P (2021). Renal tubule Cpt1a overexpression protects from kidney fibrosis by restoring mitochondrial homeostasis. J Clin Invest.

[18] Cheng S, Lu Y, Li Y, Gao L, Shen H, Song K (2018). Hydrogen sulfide inhibits epithelial-mesenchymal transition in peritoneal mesothelial cells. Sci Rep.

[19] Tran M, Tam D, Bardia A, Bhasin M, Rowe GC, Kher A (2011). PGC-1alpha promotes recovery after acute kidney injury during systemic inflammation in mice. J Clin Invest.

[20] Chau BN, Xin C, Hartner J, Ren S, Castano AP, Linn G (2012). MicroRNA-21 promotes fibrosis of the kidney by silencing metabolic pathways. Sci Transl Med.

[21] Kim SW, Yoon SJ, Chuong E, Oyolu C, Wills AE, Gupta R (2011). Chromatin and transcriptional signatures for Nodal signaling during endoderm formation in hESCs. Dev Biol.

[22] Ko YA, Mohtat D, Suzuki M, Park AS, Izquierdo MC, Han SY (2013). Cytosine methylation changes in enhancer regions of core pro-fibrotic genes characterize kidney fibrosis development. Genome Biol.

[23] Carracedo A, Cantley LC, Pandolfi PP (2013). Cancer metabolism: fatty acid oxidation in the limelight. Nat Rev Cancer.

[24] Li XX, Wang ZJ, Zheng Y, Guan YF, Yang PB, Chen X (2018). Nuclear Receptor Nur77 Facilitates Melanoma Cell Survival under Metabolic Stress by Protecting Fatty Acid Oxidation. Mol Cell.

[25] Jiang J, Wang K, Chen Y, Chen H, Nice EC, Huang C (2017). Redox regulation in tumor cell epithelial-mesenchymal transition: molecular basis and therapeutic strategy. Signal Transduct Target Ther.

[26] Zhang J, Chen Y, Chen T, Miao B, Tang Z, Hu X (2021). Single-cell transcriptomics provides new insights into the role of fibroblasts during peritoneal fibrosis. Clin Transl Med.

[27] Hue L, Taegtmeyer H (2009). The Randle cycle revisited: a new head for an old hat. Am J Physiol Endocrinol Metab.

[28] Fondevila MF, Fernandez U, Heras V, Parracho T, Gonzalez-Rellan MJ, Novoa E (2022). Inhibition of carnitine palmitoyltransferase 1A in hepatic stellate cells protects against fibrosis. J Hepatol.

[29] Hung KY, Liu SY, Yang TC, Liao TL, Kao SH (2014). High-dialysate-glucose-induced oxidative stress and mitochondrial-mediated apoptosis in human peritoneal mesothelial cells. Oxid Med Cell Longev.

[30] Xie X, Wang J, Xiang S, Chen Z, Zhang X, Chen J (2019). Dialysate cell-free mitochondrial DNA fragments as a marker of intraperitoneal inflammation and peritoneal solute transport rate in peritoneal dialysis. Bmc Nephrol.

[31] Wu J, Li J, Feng B, Bi Z, Zhu G, Zhang Y (2022). Activation of AMPK-PGC-1alpha pathway ameliorates peritoneal dialysis related peritoneal fibrosis in mice by enhancing mitochondrial biogenesis. Ren Fail.

[32] Lu H, Chen W, Liu W, Si Y, Zhao T, Lai X (2020). Molecular hydrogen regulates PTEN-AKT-mTOR signaling via ROS to alleviate peritoneal dialysis-related peritoneal fibrosis. Faseb J.

[33] Ishibashi Y, Sugimoto T, Ichikawa Y, Akatsuka A, Miyata T, Nangaku M (2002). Glucose dialysate induces mitochondrial DNA damage in peritoneal mesothelial cells. Perit Dial Int.

[34] Cardanho-Ramos C, Morais VA (2021). Mitochondrial Biogenesis in Neurons: How and Where. Int J Mol Sci.

[35] Carracedo A, Weiss D, Leliaert AK, Bhasin M, de Boer VC, Laurent G (2012). A metabolic prosurvival role for PML in breast cancer. J Clin Invest.

[36] Zhao M, Wang Y, Li L, Liu S, Wang C, Yuan Y (2021). Mitochondrial ROS promote mitochondrial dysfunction and inflammation in ischemic acute kidney injury by disrupting TFAM-mediated mtDNA maintenance. Theranostics.

[37] Margetts PJ, Bonniaud P, Liu L, Hoff CM, Holmes CJ, West-Mays JA (2005). Transient overexpression of TGF-beta1 induces epithelial mesenchymal transition in the rodent peritoneum. J Am Soc Nephrol.

[38] Guo H, Leung JC, Lam MF, Chan LY, Tsang AW, Lan HY (2007). Smad7 transgene attenuates peritoneal fibrosis in uremic rats treated with peritoneal dialysis. J Am Soc Nephrol.

[39] Shi Y, Tao M, Wang Y, Zang X, Ma X, Qiu A (2020). Genetic or pharmacologic blockade of enhancer of zeste homolog 2 inhibits the progression of peritoneal fibrosis. J Pathol.

[40] Ferrantelli E, Liappas G, Vila CM, Keuning ED, Foster TL, Vervloet MG (2016). The dipeptide alanyl-glutamine ameliorates peritoneal fibrosis and attenuates IL-17 dependent pathways during peritoneal dialysis. Kidney Int.

[41] Guo Y, Wang L, Gou R, Wang Y, Shi X, Pang X (2020). SIRT1-modified human umbilical cord mesenchymal stem cells ameliorate experimental peritoneal fibrosis by inhibiting the TGF-beta/Smad3 pathway. Stem Cell Res Ther.

[42] Chen YT, Chang YT, Pan SY, Chou YH, Chang FC, Yeh PY (2014). Lineage tracing reveals distinctive fates for mesothelial cells and submesothelial fibroblasts during peritoneal injury. J Am Soc Nephrol.

[43] Yang L, Besschetnova TY, Brooks CR, Shah JV, Bonventre JV (2010). Epithelial cell cycle arrest in G2/M mediates kidney fibrosis after injury. Nat Med.

[44] Cheng S, Wang G, Wang Y, Cai L, Qian K, Ju L (2019). Fatty acid oxidation inhibitor etomoxir suppresses tumor progression and induces cell cycle arrest via PPARgamma-mediated pathway in bladder cancer. Clin Sci (Lond).

[45] Lionetti V, Stanley WC, Recchia FA (2011). Modulating fatty acid oxidation in heart failure. Cardiovasc Res.

[46] Schmidt-Schweda S, Holubarsch C (2000). First clinical trial with etomoxir in patients with chronic congestive heart failure. Clin Sci (Lond).

[47] Bristow M (2000). Etomoxir: a new approach to treatment of chronic heart failure. Lancet.

[48] Tan Z, Xiao L, Tang M, Bai F, Li J, Li L (2018). Targeting CPT1A-mediated fatty acid oxidation sensitizes nasopharyngeal carcinoma to radiation therapy. Theranostics.

