# Supplementary material for: Restoration of CPT1A-mediated fatty acid oxidation in mesothelial cells protects against peritoneal fibrosis
Source: Theranostics. 2023 Aug 15;13(13):4482–96. doi: 10.7150/thno.84921 (PMC10465225; doi:10.7150/thno.84921)
Supplement: Supplementary file 1 — Supplementary methods, figures and tables. [file thnov13p4482s1.pdf]

Restoration of CPT1A-mediated fatty acid oxidation in mesothelial cells protects against peritoneal fibrosis

Wenyan Su, Zuoyu Hu, Xiaohong Zhong, Ansheng Cong, Ying Zhang, Zhanmei Zhou, Jianyi Li, Cailing Su, Yujie Huang, Wei Cao

Supplementary material Table of Contents

Supplemental Methods.

Figure S1. Heat maps showing the expression of key FAO enzymes in cell clusters of normal and LT-PD groups in scRNA-seq data

Figure S2. The expression of fibrotic markers and profibrogenic transcription factors in human mesothelial cells in scRNA-seq data

Figure S3. Expression of profibrogenic transcription factors in mesothelial cells from LT-PD patients and mesothelial cells from PD mice

Figure S4. TGF-β1 induces profibrotic phenotype and mitochondrial dysfunction, and increases apoptosis in primary MPMCs

Figure S5. TGF-β1 decreases FAO and induces profibrotic phenotype in Met-5A cells

Figure S6. Restoration of CPT1A expression reverses TGF-β1 induced FAO suppression and profibrotic phenotype in mesothelial cells

Figure S7. Mesothelial cells treated with Mito-TEMPO reverses TGF-β1-induced mitochondrial superoxide generation and profibrotic phenotype

Figure S8. Restoration of CPT1A expression protects against fibrosis in mouse peritoneum

Figure S9. In the setting of PD, mesothelial cells display a profibrotic secretome that activates resident peritoneal fibroblasts

Table S1. Gene markers of each cell cluster

Table S2. List of siRNAs used to knock out the indicated targets

Table S3. List of primers used for real-time PCR

Supplemental Methods

Gene transfection of cultured cells

Transfection of Adenoviruses-CPT1A into primary mouse mesothelial cells

The full-length mouse CPT1A cDNA (NM_013495) was inserted into a CMV-MCS-Flag vector (GV138) using restriction sites AgeI/NheI. After sequencing ensured accuracy of the vector, adenovirus (Ad) was packaged, purified, and titrated by GeneChem. To overexpress CPT1A in primary mouse mesothelial cells, cells were seeded into 6-well plates to reach a confluence of 60%, and then infected with Ad-CPT1A (10 MOI) for 24 h.

Transfection of Lentiviruses-CPT1A into human Met-5A cells

The full-length human CPT1A cDNA (NM_001876) was inserted into a Ubi-MCS-FLAG-CBh-GFP-IRES-puromycin vector (GV492) using restriction sites BamHI/AgeI. After sequencing ensured accuracy of the vector, lentivirus (Lv) was packaged, purified, and titrated by GeneChem. To overexpress CPT1A in human Met-5A cells, cells were seeded into 6-well plates to reach a confluence of 60%, and then infected with lentivirus-CPT1A (50 MOI) for 24 h. Stable cells were established after puromycin selection.

Small-interfering RNA

The small interfering (si) RNA oligonucleotides targeted to Smad3 and PGC1α were used to downregulate Smad3 and PGC1α respectively in Met-5A cells. Cells were seeded at 1×10^5 cells per well in 6-well plates and transfected with Smad3 or PGC1a siRNA (100nM) with lipofectamine 3000 (Invitrogen, Carlsbad, CA, USA) according

to the manufacturer's instructions (GeneChem). In parallel, scrambled siRNA (100 nM) was used as negative controls. siRNA sequences are listed in Table S2.

Transfection of PPARGC1A Plasmid into human Met-5A cells

The full length human PPARGC1A (PGC1α) cDNA (NM_013261) was inserted into pcDNA3.1 vector (GeneChem) at the NheI/EcoRI sites. To overexpress PGC1α in Met-5A cells, cells were transfected with pcDNA3.1-PGC1α plasmids or pcDNA3.1 empty vector (GeneChem) with Lipofectamine 3000 (Invitrogen, Carlsbad, CA, USA) according to the manufacturer's instructions.

Peritoneal function

Peritoneal function was measured by modified peritoneal equilibration tests [1]. Before mice were killed, 3 ml of 4.25% PD fluid was intraperitoneally injected. Dialysate and blood samples were collected at 0 and 2 h of dwell time. The concentrations of glucose and urea nitrogen in the dialysate and plasma were measured using an automatic biochemical analyzer (Beckman, CA, USA). The peritoneal permeability to glucose was expressed as the ratio of glucose concentration in 2-h peritoneal dialysate to 0-h peritoneal dialysate (D/D_0). The peritoneal permeability to blood urea nitrogen was expressed as the ratio of urea nitrogen concentration in dialysate to plasma at 2 h (D/P). The net ultrafiltration was calculated by removal of 3 ml from the 2-h dialysate volume.

Peritoneum histological procedures

Under anesthesia with sodium pentobarbital (50 mg/kg body weight, i.p.), mice were transcardially perfused, first with PBS and then with 4% paraformaldehyde [2]. The parietal peritoneal tissues were isolated, embedded in paraffin, and sectioned at a thickness of 4 μm. Peritoneal fibrosis was assessed on paraffin sections processed for Masson's trichrome staining and Sirius red staining [3]. The degree of fibrosis was calculated by the thickness of fibrotic (blue-stained) area in each section [4].

Immunostaining

Immunostaining was performed as previously described [2]. Briefly, paraffin sections (4 μm), frozen sections (8 μm) or 4% paraformaldehyde-fixed cells were labeled with primary antibodies at 4 °C for 1 day and then reacted with respective secondary antibodies (Thermo Fisher Scientific, MA, USA) at 37 °C for 1 h. Sections or paraformaldehyde-fixed cells were visualized under a Leica confocal microscope (Leica TCS SP2 AOBS, IL, USA). Primary antibodies used were listed as follows: anti-Collagen I (1:400, BA0325, Boster, Wuhan, China), anti-UPK3B (1:50, ab237778), anti-CPT1A (1:50, ab128568), anti-CD45 (1:50, ab282747) (all from Abcam, Cambridge, UK), anti-GFP (1:100, A21311, Invitrogen, Carlsbad, CA, USA), anti-cytokeratin 18 (1:50, 10830-1-AP, ProteinTech, IL, USA), anti-vimentin (1:50, MAB21052) and anti-CD31 (1:50, AF3628) (all from R&D Systems, MN, USA) antibody.

Cell culture and isolation

Isolation of mouse peritoneal mesothelial cells

Mouse peritoneal mesothelial cells were derived from mesentery resected from mice with different treatments [5]. Concisely, mesentery tissues were obtained from the mouse under anesthesia with sodium pentobarbital (50 mg/kg body weight, i.p.), and then digested with 0.25% Trypsin-EDTA for 10 min at 37 °C with gentle rotation. Next, the digested solution was triturated in growth medium and centrifuged. The cell pellet was washed and resuspended in PBS containing 0.5% BSA [6]. Mesothelial cells were isolated by fluorescence-activated cell sorting using UPK3B antibody [6].

Culture of NIH 3T3 fibroblasts

NIH 3T3 fibroblast cell line (mouse, American Type Culture Collection, VA, USA) were cultured in DMEM supplemented with 10% FBS and 1% penicillin-streptomycin (all from Gibco, Grand Island, NY, USA) at 37 °C in 5% CO_2 atmosphere.

Preparation of conditioned medium from human mesothelial cells

Mesothelial cells were stimulated with TGF-β1 for 24 h. Then TGF-β1 was washed out and the cells continued in culture for 24 h. The culture medium was aspirated, centrifuged, sterile filtered (0.22 μm filter). The conditioned medium was then collected and added to serum-starved NIH 3T3 fibroblasts for 24 h [7].

Measurement of OCR and ECAR

FAO-associated oxygen consumption rate (OCR) and the extracellular acidification rate (ECAR) were determined by high-resolution respirometry with the Seahorse

Bioscience XFe96 Extracellular Flux Analyzer according to the manufacturer's instructions (Agilent Technologies, CA, USA). Cells were seeded in a XF96 cell culture microplate. On the assay day, substrate-limited medium was replaced with assay medium supplemented with 2 mM Glucose and 0.5mM carnitine for 1 h at 37 °C without CO_2. Finally, just before starting the assay, 30μL BSA or palmitate-BSA FAO substrate was added. Inhibitors and activators were used in these tests at the following concentrations: oligomycin (1.5 μM), FCCP (1 μM), rotenone/antimycin (Rot/AA, 0.5 μM). All results was determined by normalizing the measurements to cell counts by quantifying the hoechst staining in each well.

ATP measurement.

ATP content was determined in cells using the Enhanced ATP Assay Kit (S00237, Beyotome, Shanghai, China) following the manufacturer's instructions.

Mitochondrial copy number determination.

Genomic DNA was extracted from cells using the TaKaRa MiniBEST Universal Genomic DNA Extraction Kit (TaKaRa Biotechnology, Shiga, Japan) according to the manufacturer's instructions. Mitochondrial (mt) abundance was determined with the Mouse Mitochondrial DNA Copy Number Assay Kit (Detroit R&D, MI, USA). Relative mtDNA copy number was presented as the ratio of mtDNA to nuclear DNA.

Mitochondrial superoxide detection

The MitoSOX Red Mitochondrial Superoxide Indicator (Thermo Fisher Scientific, MA, USA) was utilized to detect mitochondrial superoxide, following the manufacturer's instructions and then visualized under a Leica confocal microscope.

CPT1 Activity Assay

CPT1 activity was assessed with CPT1 Activity Assay Kit (Suzhou Comin Biotechnology Company) according to the manufacturer's instructions [8]. CPT1 enzymatic activity was detected by measuring the release of CoA-SH from palmitoyl-CoA using the general thiol reagent DTNB. Briefly, samples were separately mixed with reaction buffer containing 1 mM EDTA, 200 μM 5, 5′-dithiobis-(2-nitrobenzoic acid) (DTNB), and 80 μM palmitoyl-CoA. After 5-min pre-incubation at 30 °C, the reaction was initiated with the supplement of L-carnitine, followed by an immediate photometric measurement at 412 nm. As blank control, the L-carnitine was substituted for sterilized distilled water. The calculation of CPT1 activity was based on absorbance and normalized with protein concentrations.

TGF-β1 and CTGF measurement by enzyme-linked immunosorbent assay (ELISA)

TGF-β1 concentrations in effluent of PD mice and supernatants of cultured mesothelial cells were detected using ELISA kits (R&D System) according to manufacturer's instruction. CTGF concentrations in supernatants of cultured mesothelial cells were detected using ELISA kit (Abcam) according to manufacturer's

instruction.

Measurement of cellular NADPH levels

The NADPH levels in cultured cells were measured using an Amplite™ Fluorimetric NADPH Assay Kit (AAT Bioquest, Sunnyvale, CA, USA) according to manufacturer's instruction [9].

Luciferase reporter assay

To detect whether CPT1A is transcribed by PGC1α, we cloned the CPT1A promoter sequence between -2015 to +15 into pGL3-basic vector (GeneChem). A luciferase reporter pGL3-CPT1A-promoter (Luc-CPT1A), pRL-TK vector (GeneChem), PGC1α-overexpressing vector (GeneChem) were transfected into cultured cells with Lipofectamine 3000 (Invitrogen) for 48 h according to the manufacturer's recommendations. Luciferase activities were detected by the Dual-Luciferase Reporter Assay System (E1910, Promega). Luciferase activity was normalized to Renilla luciferase activity [10].

CCK8 assay

Cells were seeded into 96-well plates at a density of 3 × 103 cells per well. Cell viability was measured at the indicated time points, using a Cell Counting Kit (CCK8, Dojindo, Japan) according to the manufacturer's instructions. Absorbance at 450 nm was measured using a multifunctional microplate reader (Bio-Rad Laboratories,

Hercules, CA).

Western blot analysis

Lysates of tissues or cells were prepared, and western blots were performed [11] with the following primary antibodies: anti-collagen I (1:500, BA0325, Boster), anti-fibronectin (1:1000, 15613-1-AP), anti-PGC1α (1:2000, 66369-1-lg), anti-Caspase 3 (1:1000, 19677-1-AP), anti-Snail (1:1000, 13099-1-AP), anti-Slug (1:1000, 12129-1-AP), anti-ZEB2 (1:1000, 67514-1-lg) (all from ProteinTech), anti-E-cadherin (1:1000, 3195S), anti-cleaved caspase 3 (1:1000, 9664S) (all from Cell Signaling Technology, MA, USA), anti-CPT1A (1:1000, ab128568), anti-Smad3 (1:1000, ab40854) (all from Abcam) antibody, followed by specific secondary antibodies (LICOR, NE, USA). β-actin antibody (1:1000, GTX109639, GeneTex) was used as loading controls. Band densitometry was performed using Image J software (NIH, USA).

RNA extraction and Real-time PCR

Total RNA was isolated from cells or tissues with TRIzol reagent (Invitrogen), or RNeasy Plus Micro Kit (Qiagen, Hilden, Germany). Real-time PCR was performed using TaKaRa SYBR® Premix Ex Taq^{TM} II kit (Takara Biotechnology), with β-Actin serving as the internal control. The primers used were listed in Table S3.

References

1. Shi Y, Tao M, Wang Y, Zang X, Ma X, Qiu A, et al. Genetic or pharmacologic blockade of enhancer of zeste homolog 2 inhibits the progression of peritoneal fibrosis. J Pathol. 2020; 250: 79-94.
2. Cao W, Wu L, Zhang X, Zhou J, Wang J, Yang Z, et al. Sympathetic Overactivity in CKD Disrupts Buffering of Neurotransmission by Endothelium-Derived Hyperpolarizing Factor and Enhances Vasoconstriction. J Am Soc Nephrol. 2020; 31: 2312-25.
3. De Vriese AS, Tilton RG, Mortier S, Lameire NH. Myofibroblast transdifferentiation of mesothelial cells is mediated by RAGE and contributes to peritoneal fibrosis in uraemia. Nephrol Dial Transplant. 2006; 21: 2549-55.
4. Ferrantelli E, Liappas G, Vila CM, Keuning ED, Foster TL, Vervloet MG, et al. The dipeptide alanyl-glutamine ameliorates peritoneal fibrosis and attenuates IL-17 dependent pathways during peritoneal dialysis. Kidney Int. 2016; 89: 625-35.
5. Cheng S, Lu Y, Li Y, Gao L, Shen H, Song K. Hydrogen sulfide inhibits epithelial-mesenchymal transition in peritoneal mesothelial cells. Sci Rep. 2018; 8: 5863.
6. Si M, Wang Q, Li Y, Lin H, Luo D, Zhao W, et al. Inhibition of hyperglycolysis in mesothelial cells prevents peritoneal fibrosis. Sci Transl Med. 2019; 11: eaav5341.
7. Yang L, Besschetnova TY, Brooks CR, Shah JV, Bonventre JV. Epithelial cell

cycle arrest in G2/M mediates kidney fibrosis after injury. Nat Med. 2010; 16: 535-43.

8. Hao F, Tian M, Zhang X, Jin X, Jiang Y, Sun X, et al. Butyrate enhances CPT1A activity to promote fatty acid oxidation and iTreg differentiation. Proc Natl Acad Sci U S A. 2021; 118: e2014681118.
9. Pike LS, Smift AL, Croteau NJ, Ferrick DA, Wu M. Inhibition of fatty acid oxidation by etomoxir impairs NADPH production and increases reactive oxygen species resulting in ATP depletion and cell death in human glioblastoma cells. Biochim Biophys Acta. 2011; 1807: 726-34.
10. Du Q, Tan Z, Shi F, Tang M, Xie L, Zhao L, et al. PGC1alpha/CEBPB/CPT1A axis promotes radiation resistance of nasopharyngeal carcinoma through activating fatty acid oxidation. Cancer Sci. 2019; 110: 2050-62.
11. Shi H, Qi H, Xie D, Zhuang J, Qi H, Dai Y, et al. Inhibition of ACSF2 protects against renal ischemia/reperfusion injury via mediating mitophagy in proximal tubular cells. Free Radical Bio Med. 2023; 198: 68-82.

Figure legends

Figure S1. Heat maps showing the expression of key FAO enzymes in cell clusters of normal and LT-PD groups in scRNA-seq data

(A) Uniform manifold approximation and projection (UMAP) shows single-cell populations from the normal peritoneum (normal, n = 3) and from effluent of patients on long-term PD (LT-PD, n = 4) separately using scRNA-seq data GSE130888. (B-H) Key FAO enzymes in monocyte (B), T cell (C), NK cell (D), B cell (E), endothelial cell (F), fibroblast (G) and myofibroblast (H) from normal and LT-PD groups.

Figure S2. The expression of fibrotic markers and profibrogenic transcription factors in human mesothelial cells in scRNA-seq data

(A) UMAP plots demonstrating the expression of COL1A1 in the eight cell clusters from LT-PD group. The color gradient is based on the normalized gene expression. Myofibro: Myofibroblast; Fibro: Fibroblast; EC: Endothelial cell. (B) UMAP plots demonstrating the expression of VIM, FN1, ACTA2, PDGFRA and PDGFRB in mesothelial cell population selected from A. The color gradient is based on the normalized gene expression. (C) Violin plots demonstrating the expression of profibrogenic transcription factors in mesothelial cells derived from effluents of patients on LT-PD and the normal peritoneum. The y axis shows the normalized gene expression. (D) Ordering of scRNA-seq expression data of mesothelial cells according to the pseudotime produced by Monocle analysis.

Figure S3. Expression of profibrogenic transcription factors in mesothelial cells from LT-PD patients and mesothelial cells from PD mice

(A) The schematic diagram of sorting mesothelial cells by fluorescence-activated cell sorting using UPK3B antibody from normal peritoneum and from effluents of patients on LT-PD. (B) mRNA levels of CPT1A, CPT1B and CPT1C in HPMCs isolated from normal human peritoneum (n = 6). Unpaired *t* test, **P* < 0.05 *versus* CPT1A levels. (C) Protein levels of ZEB2, Snail and Slug in HPMCs isolated from normal and LT-PD group. Unpaired *t* test, **P* < 0.05 *versus* normal group. (D) Peritoneal fibrosis presented by Sirius Red staining in mice treated with saline or PDF. Scale bar, 100 μm. (E) The peritoneal ultrafiltration volume of mice treated with saline or PDF. Unpaired *t* test, **P* < 0.05 *versus* saline group. (F) Quantitative data showing CPT1A expression on MPMCs in Figure 2E. Unpaired *t* test, **P* < 0.05 *versus* saline group. (G) CPT1 activity in isolated MPMCs. Unpaired *t* test, **P* < 0.05 *versus* saline group. (H) Protein levels of ZEB2, Snail and Slug in isolated MPMCs. Unpaired *t* test, **P* < 0.05 *versus* saline group. (I) mRNA levels of mitochondrial biogenesis-associated genes in isolated MPMCs. Unpaired *t* test, **P* < 0.05 *versus* saline group. (J) TGF-β1 levels in peritoneal effluent and peritoneal tissues from mice treated with saline or PDF. Unpaired *t* test, **P* < 0.05 *versus* saline group. Data expressed as mean ± SD in B-C and E-J (n = 6 in each group).

Figure S4. TGF-β1 induces profibrotic phenotype and mitochondrial dysfunction, and increases apoptosis in primary MPMCs

(A) Immunofluorescence staining for cytokeratin-18, vimentin, CD45 and CD31 in primary MPMCs. Scale bar, 100 μm. (B) Protein levels of ZEB2, Snail and Slug in primary MPMCs treated with or without TGF-β1. Unpaired *t* test, **P* < 0.05 *versus* control. (C) mRNA levels of Fn1 and Col1a1 in primary MPMCs treated with indicated concentrations of TGF-β1. One-way ANOVA with Bonferroni correction, **P* < 0.05 *versus* control. (D) mRNA level of Cpt1a in primary MPMCs treated with indicated concentrations of TGF-β1. One-way ANOVA with Bonferroni correction, **P* < 0.05 *versus* control. (E) mtDNA copy number was determined in primary MPMCs treated with or without TGF-β1. Unpaired *t* test, **P* < 0.05 *versus* control. (F) mRNA levels of apoptosis markers in primary MPMCs treated with or without TGF-β1. Unpaired *t* test, **P* < 0.05 *versus* control. Data expressed as mean ± SD in B-F (n = 6 in each group).

Figure S5. TGF-β1 decreases FAO and induces profibrotic phenotype in Met-5A cells

(A) Protein levels of E-cad, FN and Co I in Met-5A cells treated with or without TGF-β1. Unpaired *t* test, **P* < 0.05 *versus* control. (B) Transcript levels of key FAO enzymes in Met-5A cells treated with or without TGF-β1. Unpaired *t* test, **P* < 0.05 *versus* control. (C) CPT1A protein level in Met-5A cells treated with or without TGF-β1. Unpaired *t* test, **P* < 0.05 *versus* control. (D) OCR measurement of Mito Stress assay in Met-5A cells. Where indicated, cells are pretreated with palmitate-BSA FAO substrate (Palm:BSA, 30 μL) or the CPT1 inhibitor etomoxir

(Eto, 4 μM). One-way ANOVA with Bonferroni correction, *P < 0.05. (E) OCR measurement in Met-5A cells treated with or without TGF-β1. Unpaired t test, *P < 0.05 *versus* control. (F) Quantification of ECAR measurement in E. unpaired t test, *P < 0.05 *versus* control. Data expressed as mean ± SD in A-F (n = 6 in each group).

Figure S6. Restoration of CPT1A expression reverses TGF-β1 induced FAO suppression and profibrotic phenotype in mesothelial cells

(A-E) Primary MPMCs are transfected with either Ad-CPT1A or Ad-NC. Real-time PCR showing upregulation of CPT1A in MPMCs transfected with Ad-CPT1A (A). Unpaired t test, *P < 0.05 *versus* Ad-NC. MPMCs overexpressing CPT1A are protected from TGF-β1-induced detrimental changes, including levels of profibrogenic transcription factors (B), NADPH production (C), and expression of mitochondrial biogenesis-associated genes (D) and apoptosis marker C-casp3 (E). One-way ANOVA with Bonferroni correction, *P < 0.05. (F-H) Met-5A cells are transfected with either Lv-CPT1A or Lv-NC. Immunoblots showing upregulation of CPT1A in Met-5A cells transfected with Lv-CPT1A (F). Unpaired t test, *P < 0.05 *versus* Lv-NC. Overexpression of CPT1A in Met-5A cells reverses the ability of TGF-β1 to reduce OCR (G), and its ability to downregulate E-cad and upregulate FN and Co I (H). One-way ANOVA with Bonferroni correction, *P < 0.05. Data expressed as mean ± SD in A-H (n = 6 in each group).

Figure S7. Mesothelial cells treated with Mito-TEMPO reverses TGF-β1-induced

mitochondrial superoxide generation and profibrotic phenotype

(A-C) MPMCs treated with Mito-TEMPO are protected from TGF-β1-induced detrimental changes, including mitochondrial superoxide generation (A; Scale bar, 50 μm), and expression of profibrogenic transcription factors (B) and profibrotic markers (C). One-way ANOVA with Bonferroni correction, $^*P < 0.05$. (D) CPT1A overexpression abolishes the protective responses induced by Mito-TEMPO in TGFβ1-treated MPMCs. One-way ANOVA with Bonferroni correction, $^*P < 0.05$. ns, no significance. Data expressed as mean ± SD in B-D (n = 6 in each group).

Figure S8. Restoration of CPT1A expression protects against fibrosis in mouse peritoneum

(A-E) Restoration of CPT1A in mesothelial cells in PD mice is achieved by injection of AAV-CPT1A-GFP (CPT1A OE) into peritoneal cavity of mice 21 days before daily PDF treatment. Peritoneal fibrosis presented by Sirius Red staining (A; Scale bar, 100 μm). The peritoneal ultrafiltration volume of mice examined by modified peritoneal equilibration test (B). Protein levels of profibrogenic transcription factors ZEB2, Snail and Slug in isolated MPMCs (C). Protein levels of C-casp3 and casp3 in isolated MPMCs (D). Transcript levels of glycolysis-related genes in isolated MPMCs (E). One-way ANOVA with Bonferroni correction, $^*P < 0.05$. (F-J) Pharmacological improvement of FAO is achieved by intraperitoneal injection of mice with C75 three times per week starting 1 day after PDF treatment. Body weight, serum creatinine (Scr), Alanine aminotransferase (ALT) and Aspartate aminotransferase (AST) in mice

(F). CPT1 activity in isolated MPMCs (G). Peritoneal fibrosis presented by Sirius Red staining (H; Scale bar, 100 μm). The peritoneal ultrafiltration volume of mice examined by modified peritoneal equilibration test (I). Transcript levels of glycolysis-related genes in isolated MPMCs (J). One-way ANOVA with Bonferroni correction in F, G and I; Kruskal-Wallis test in J, *$P < 0.05$. ns, no significance. (K and L) Pharmacological inhibition of FAO is achieved by intraperitoneally injecting mice with etomoxir three times per week starting 1 day after PDF treatment. Peritoneal fibrosis presented by Sirius Red staining. (K; Scale bar, 100 μm). The peritoneal ultrafiltration volume of mice examined by modified peritoneal equilibration test (L). One-way ANOVA with Bonferroni correction, *$P < 0.05$. Data expressed as mean ± SD in B-G, I and L; Data expressed as median with IQR in J (n = 6 in each group).

Figure S9. In the setting of PD, mesothelial cells display a profibrotic secretome that activates resident peritoneal fibroblasts

(A) Violin plots demonstrating the expression of TGF-β1 and CTGF in human mesothelial cells derived from the normal and LT-PD group in scRNA-seq data. (B-D) TGF-β1 treatment of primary MPMCs (5 ng/mL, 24 h) upregulates the expression of fibrogenic growth factors TGF-β1 and CTGF in both cell homogenates and supernatants (B). The conditioned medium from TGF-β1-treated mesothelial cells (TGF-β1-CM) enhances cell viability (C, measured by CCK-8 assay) and production of Fn, Co I and α-SMA (D) in fibroblasts. Transfection of MPMCs with Ad-CPT1A

(*versus* Ad-NC) reverses all the TGF-β1-induced effects. One-way ANOVA with Bonferroni correction, *$P < 0.05$. Data expressed as mean ± SD in B-D (n = 6 in each group).

Table S1 (separate file). Gene markers of each cell cluster

Table S2. List of siRNAs used to knock out the indicated targets

Target gene	sense	antisense
SMAD3	GCGUGAAUCCCUACCACUATT	UAGUGGUAGGGAUUCACGCAG
PPARGC1A	GCUCCAAGACUCUAGACAATT	UUGUCUAGAGUCUUGGAGCTT

Table S3. List of primers used for real-time PCR

Gene	Forward (5'->3')	Reverse (5'->3')
CPT1A	CGGTTGCTGATGACGGCTATGG	TGCCTTCCAAAGCGATGAGAATCC
CPT1B	CGCCGTAAACTGGACCGTGAAG	GATCCGAGTGGTGTTGAACATCCTC
CPT1C	GGTTCAGAGGGTCAGGGAAGGAG	CAGAAGTCGGTGGATGTCATTGAGG
ACOX1	GAGGGAGTTTGGCATCGCTGAC	GTGAAGCAAGGTGGGCAGGAAC
ACADVL	AGCCTCCATCCGAACCTCTGC	GCTGGATCTGTAACTGGTGTCTT
ACADM	GCTGGCTCGGATGGCTATTCTG	TTTGCTGATGGCGGCTTCTATCTG
ACTB	TGGCACCCAGCACAATGAA	CTAAGTCATAGTCCGCCTAGAAGCA
Cpt1a	CTCTGCTGCATGGTAGATGTT	GCTCTGCGTTTATGCCTATCT
Acox1	CTTGGATGGTAGTCCGGAGA	TGGCTTCGAGTGAGGAAGTT
Acadvl	GCTGGCTCGGATGGCTATTCTG	TTTGCTGATGGCGGCTTCTATCTG
Acadm	AATTACCGAAGAGTTGGCGTATGGG	ACATCATTGGCTGCTCCGTCATC
Nrf1	AGAAACGGAAACGGCCTCAT	ACTCATCCAACGTGGCTCTG
Tfam	CCCGGCAGAGACGGTTAAAA	TCCCTGAGCCGAATCATCCT
Esrra	CCAGGCTTCTCCTCACTGTC	GCCCCCTCTTCATCTAGGAC
Bcl2	CCGTCGTGACTTCGCAGAGATG	ATCCCTGAAGAGTTCCTCCACCAC
Bax	GCTACAGGGTTTCATCCAGGATCG	TGCTGTCCAGTTCATCTCCAATTCG
Apaf1	ATGGAACGGTGAAGGTGTGGAATG	GGTCGCATCAGAAGAGATAGCACAG
Glut1	GATGAAAGAAGAGGGTCGGCAGATG	CAGCACCACAGCGATGAGGATG
Hk1	ATGTGGTGGCTGTGGTCAA	ATCTCCTCCATGTAGCAGGC
Pfkm	GTTCAGAGTGGAGCGACTTG	TGCCACAGAAGTCATTGTCG
Pkm	ACCATCAAGAATGTCCGTGAAGCC	GTATCCAGAGCCACCGCAACAG
Ldha	CGCAGACAAGGAGCAGTGGAAG	ACCCGCCTAAGGTTCTTCATTATGC
Fn1	ACAAGGTTCGGGAAGAGGTT	CCGTGTAAGGGTCAAAGCAT
Col1a1	GACAGGCGAACAAGGTGACAGAG	CAGGAGAACCAGGAGAACCAGGAG
Tgfb1	CAACAATTCCTGGCGTTACCTTGG	TGTATTCCGTCTCCTTGGTTCAGC
Ctgf	AAGGACCGCACAGCAGTTGG	GCAGTTGGCTCGCATCATAGTTG
Actb	CATCCGTAAAGACCTCTATGCCAAC	ATGGAGCCACCGATCCACA

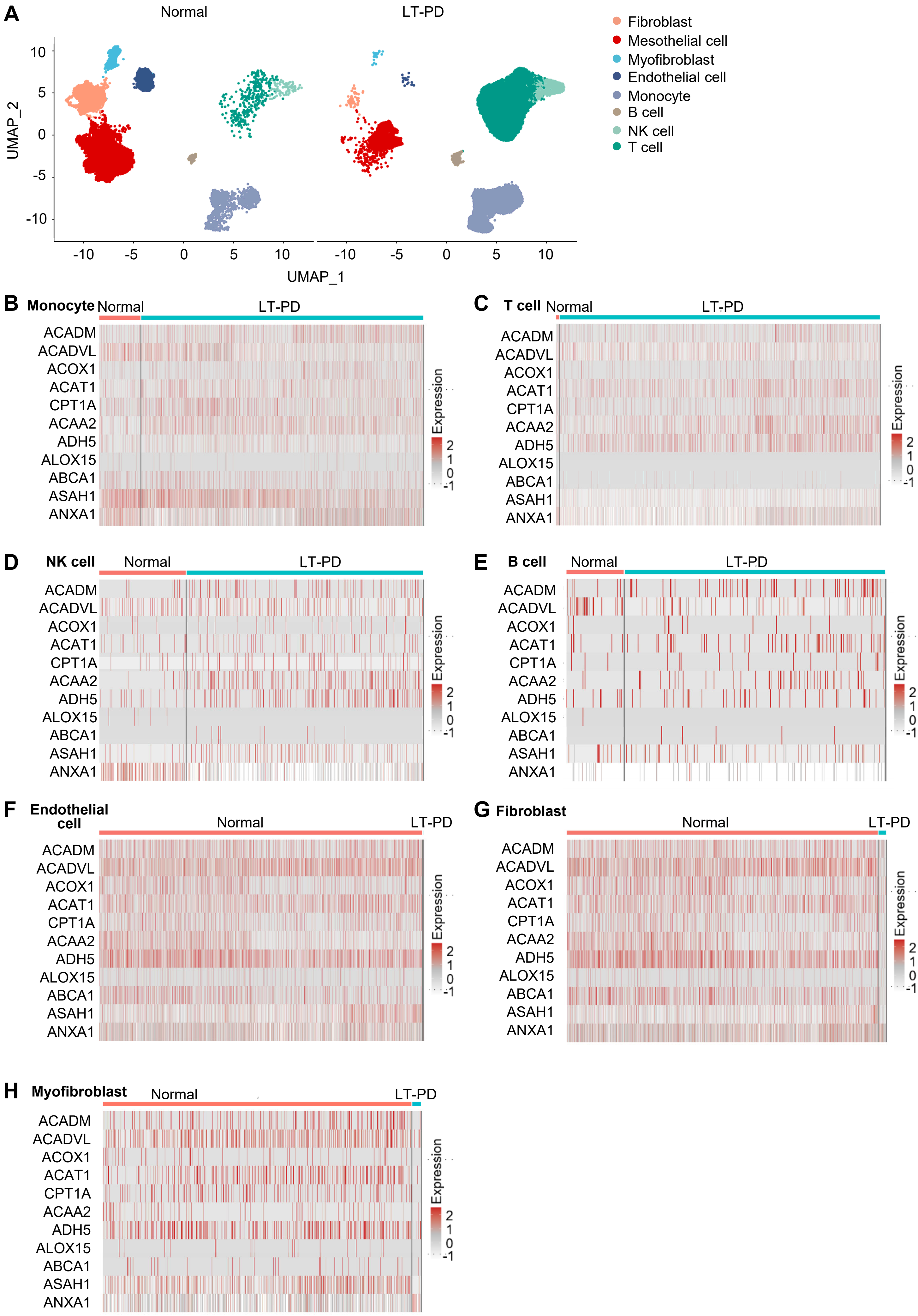
A
Normal
LT-PD
Fibroblast
Mesothelial cell
Myofibroblast
Endothelial cell
Monocyte
B cell
NK cell
T cell
UMAP_2
UMAP_1
B Monocyte
C T cell
D NK cell
E B cell
F Endothelial cell
G Fibroblast
H Myofibroblast
ACADM
ACADVL
ACOX1
ACAT1
CPT1A
ACAA2
ADH5
ALOX15
ABCA1
ASAH1
ANXA1
Expression

Figure S1. Heat maps showing the expression of key FAO enzymes in cell clusters of normal and LT-PD groups in scRNA-seq data

(A) Uniform manifold approximation and projection (UMAP) shows single-cell populations from the normal peritoneum (normal, n = 3) and from effluent of patients on long-term PD (LT-PD, n = 4) separately using scRNA-seq data GSE130888. (B-H) Key FAO enzymes in monocyte (B), T cell (C), NK cell (D), B cell (E), endothelial cell (F), fibroblast (G) and myofibroblast (H) from normal and LT-PD groups.

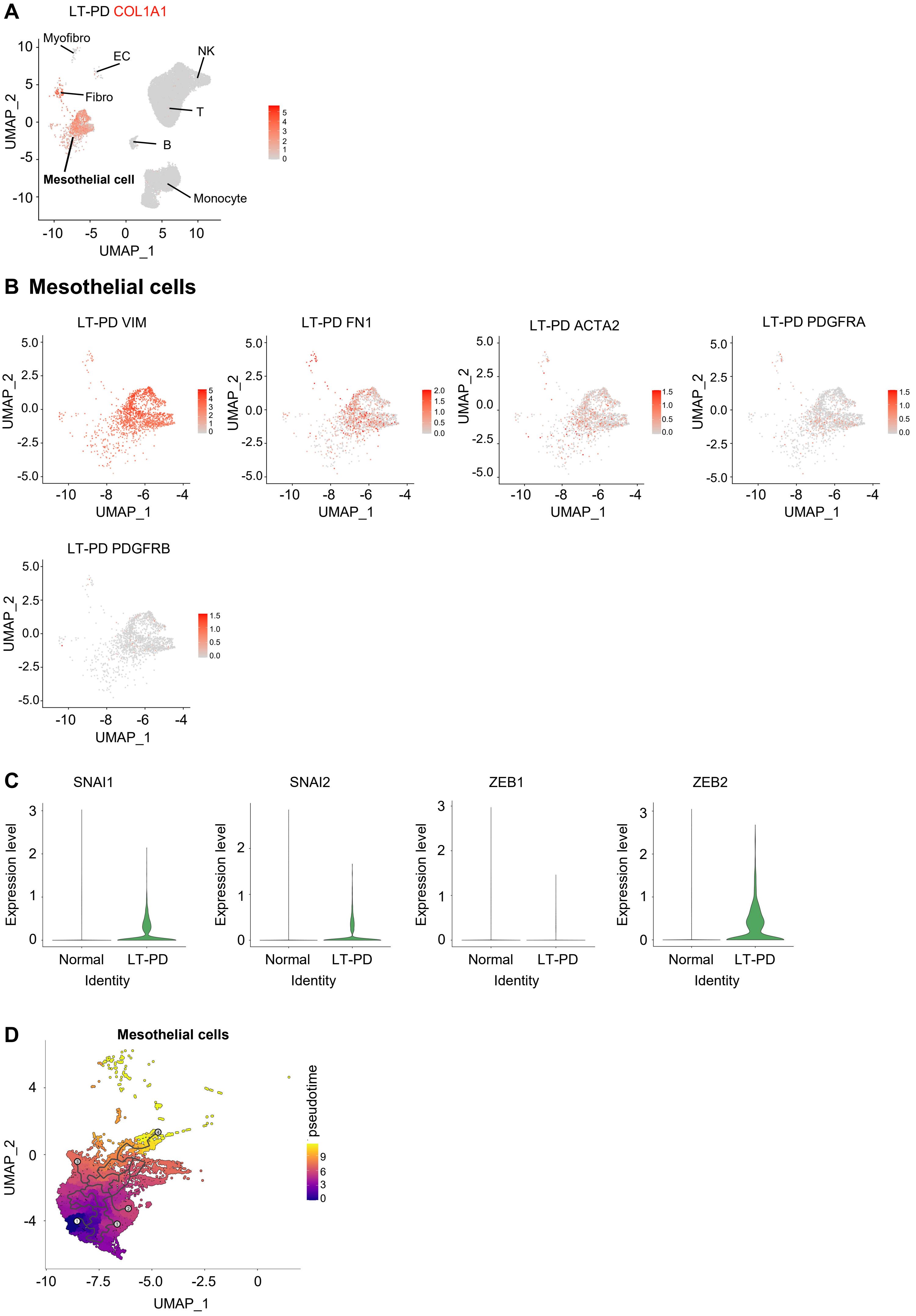
A
LT-PD COL1A1
Myofibro
EC
NK
Fibro
T
B
Mesothelial cell
Monocyte
UMAP_1
UMAP_2
B Mesothelial cells
LT-PD VIM
LT-PD FN1
LT-PD ACTA2
LT-PD PDGFRA
LT-PD PDGFRB
UMAP_1
UMAP_2
C
SNAI1
SNAI2
ZEB1
ZEB2
Expression level
Normal
LT-PD
Identity
D
Mesothelial cells
pseudotime
UMAP_1
UMAP_2

Figure S2. The expression of fibrotic markers and profibrogenic transcription factors in human mesothelial cells in scRNA-seq data

(A) UMAP plots demonstrating the expression COL1A1 in the eight cell clusters from LT-PD group. The color gradient is based on the normalized gene expression. Myofibro: Myofibroblast; Fibro: Fibroblast; EC: Endothelial cell. (B) UMAP plots demonstrating the expression of VIM, FN1, ACTA2, PDGFRA and PDGFRB in mesothelial cell population selected from A. The color gradient is based on the normalized gene expression. (C) Violin plots demonstrating the expression of profibrogenic transcription factors in mesothelial cells derived from effluents of patients on LT-PD and the normal peritoneum. The y axis shows the normalized gene expression. (D) Ordering of scRNA-seq expression data of mesothelial cells according to the pseudotime produced by Monocle analysis.

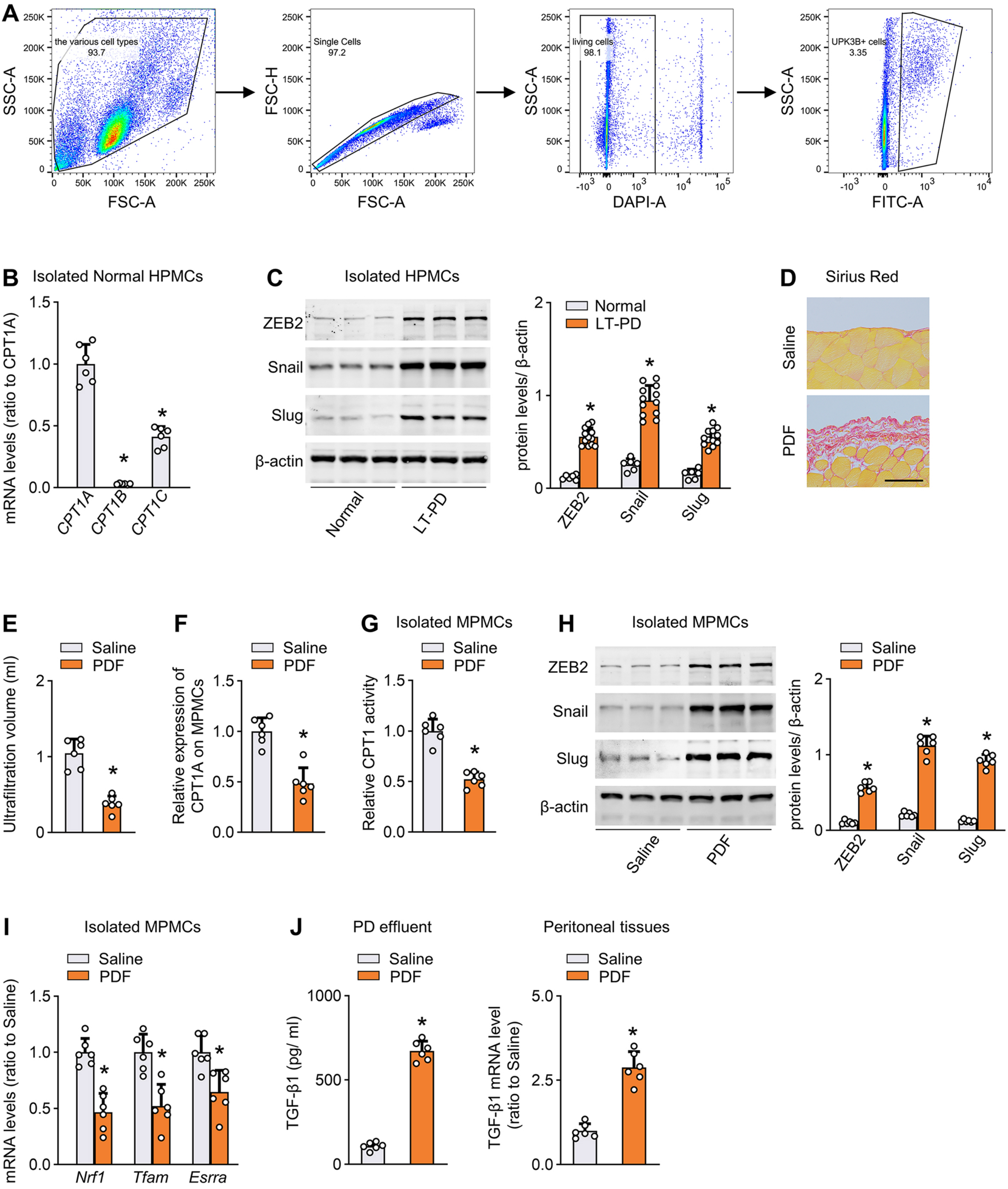

A
the various cell types 93.7
SSC-A
FSC-A
Single Cells 97.2
FSC-H
FSC-A
living cells 98.1
SSC-A
DAPI-A
UPK3B+ cells 3.35
SSC-A
FITC-A
B Isolated Normal HPMCs
mRNA levels (ratio to CPT1A)
CPT1A
CPT1B
CPT1C
C Isolated HPMCs
ZEB2
Snail
Slug
β-actin
Normal
LT-PD
protein levels/ β-actin
D Sirius Red
Saline
PDF
E
Saline
PDF
Ultrafiltration volume (ml)
F
Relative expression of CPT1A on MPMCs
G Isolated MPMCs
Relative CPT1 activity
H Isolated MPMCs
I Isolated MPMCs
mRNA levels (ratio to Saline)
Nrf1
Tfam
Esrra
J PD effluent
TGF-β1 (pg/ ml)
Peritoneal tissues
TGF-β1 mRNA level (ratio to Saline)

Figure S3. Expression of profibrogenic transcription factors in mesothelial cells from LT-PD patients and mesothelial cells from PD mice

(A) The schematic diagram of sorting mesothelial cells by fluorescence-activated cell sorting using UPK3B antibody from normal peritoneum and from effluents of patients on LT-PD. (B) mRNA levels of CPT1A, CPT1B and CPT1C in HPMCs isolated from normal human peritoneum (n = 6). Unpaired t test, *P < 0.05 versus CPT1A levels. (C) Protein levels of ZEB2, Snail and Slug in HPMCs isolated from normal and LT-PD group. Unpaired t test, *P < 0.05 versus normal group. (D) Peritoneal fibrosis presented by Sirius Red staining in mice treated with saline or PDF. Scale bar, 100 μm. (E) The peritoneal ultrafiltration volume of mice treated with saline or PDF. Unpaired t test, *P < 0.05 versus saline group. (F) Quantitative data showing CPT1A expression on MPMCs in Figure 2E. Unpaired t test, *P < 0.05 versus saline group. (G) CPT1 activity in isolated MPMCs. Unpaired t test, *P < 0.05 versus saline group. (H) Protein levels of ZEB2, Snail and Slug in isolated MPMCs. Unpaired t test, *P < 0.05 versus saline group. (I) mRNA levels of mitochondrial biogenesis-associated genes in isolated MPMCs. Unpaired t test, *P < 0.05 versus saline group. (J) TGF-β1 levels in peritoneal effluent and peritoneal tissues from mice treated with saline or PDF. Unpaired t test, *P < 0.05 versus saline group. Data expressed as mean ± SD in B-C and E-J (n = 6 in each group).

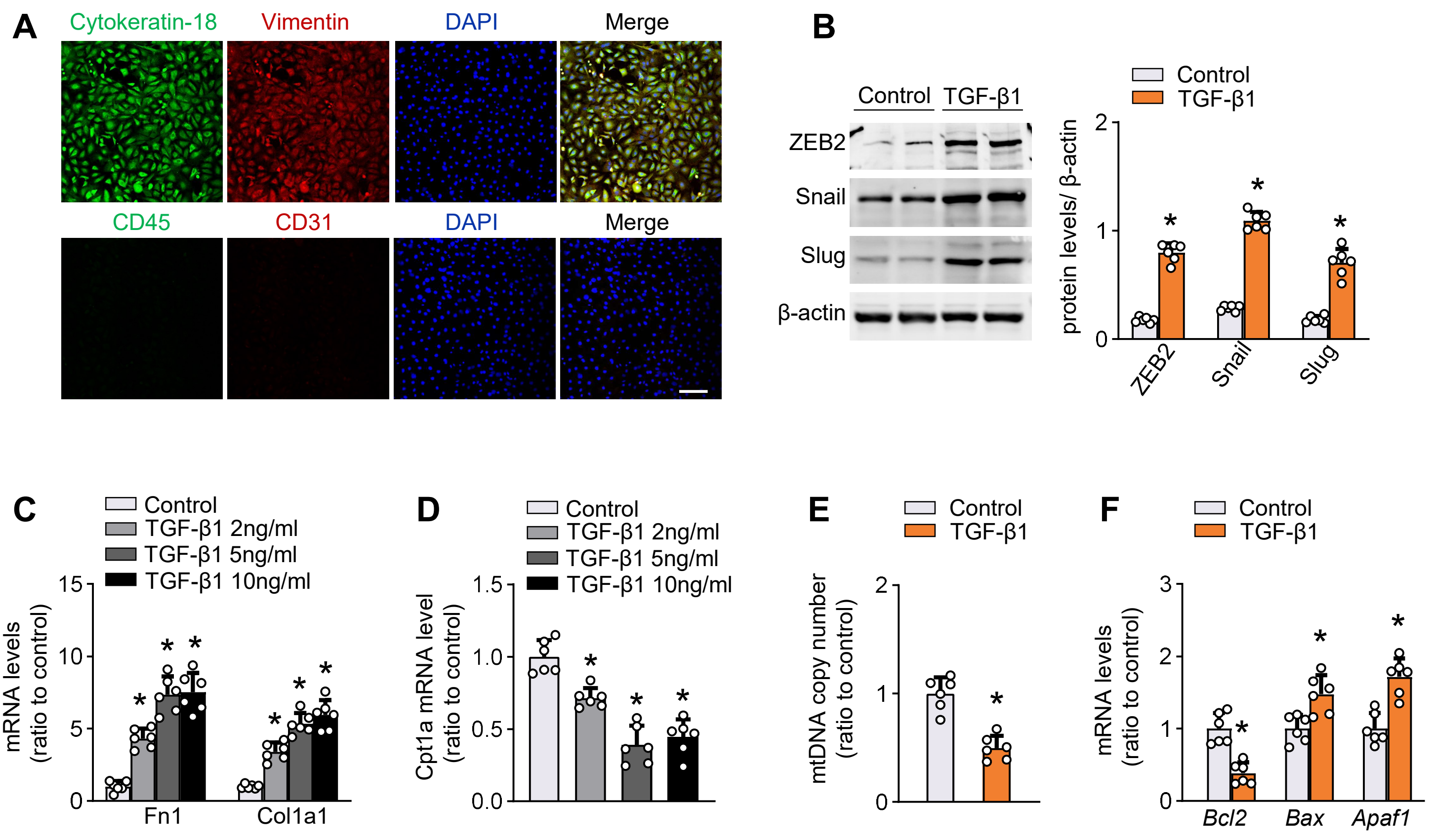

Figure S4. TGF-β1 induces profibrotic phenotype and mitochondrial dysfunction, and increases apoptosis in primary MPMCs

(A) Immunofluorescence staining for cytokeratin-18, vimentin, CD45 and CD31 in primary MPMCs. Scale bar, 100 μm. (B) Protein levels of ZEB2, Snail and Slug in primary MPMCs treated with or without TGF-β1. Unpaired t test, *P < 0.05 versus control. (C) mRNA levels of Fn1 and Col1a1 in primary MPMCs treated with indicated concentrations of TGF-β1. One-way ANOVA with Bonferroni correction, *P < 0.05 versus control. (D) mRNA level of Cpt1a in primary MPMCs treated with indicated concentrations of TGF-β1. One-way ANOVA with Bonferroni correction, *P < 0.05 versus control. (E) mtDNA copy number was determined in primary MPMCs treated with or without TGF-β1. Unpaired t test, *P < 0.05 versus control. (F) mRNA levels of apoptosis markers in primary MPMCs treated with or without TGF-β1. Unpaired t test, *P < 0.05 versus control. Data expressed as mean ± SD in B-F (n = 6 in each group).

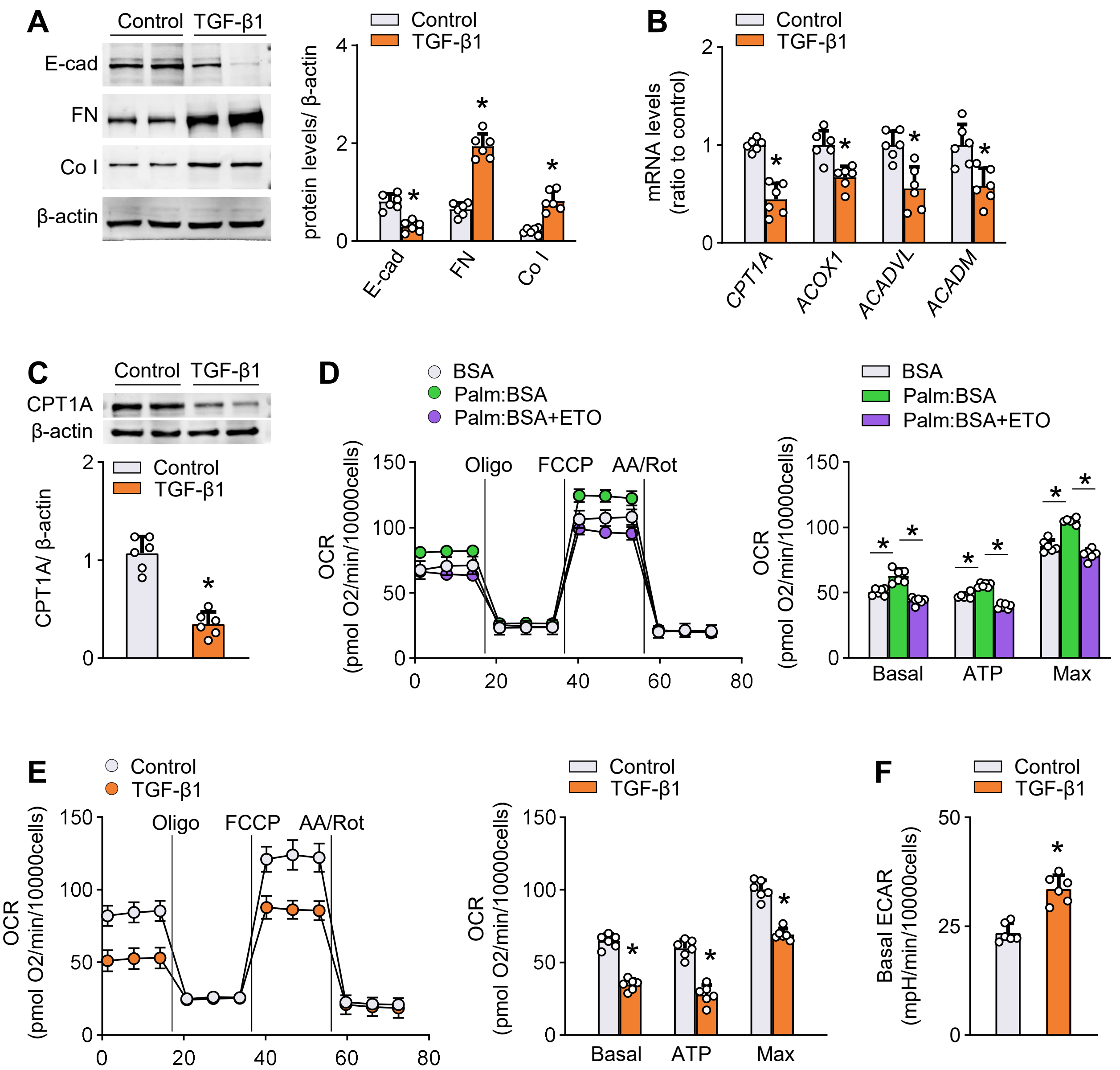

Figure S5. TGF-β1 decreases FAO and induces profibrotic phenotype in Met-5A cells

(A) Protein levels of E-cad, FN and Co I in Met-5A cells treated with or without TGF-β1. Unpaired t test, $^*P < 0.05$ versus control. (B) Transcript levels of key FAO enzymes in Met-5A cells treated with or without TGF-β1. Unpaired t test, $^*P < 0.05$ versus control. (C) CPT1A protein level in Met-5A cells treated with or without TGF-β1. Unpaired t test, $^*P < 0.05$ versus control. (D) OCR measurement of Mito Stress assay in Met-5A cells. Where indicated, cells are pretreated with palmitate-BSA FAO substrate (Palm:BSA, 30 μL) or the CPT1 inhibitor etomoxir (Eto, 4 μM). One-way ANOVA with Bonferroni correction, $^*P < 0.05$. (E) OCR measurement in Met-5A cells treated with or without TGF-β1. Unpaired t test, $^*P < 0.05$ versus control. (F) Quantification of ECAR measurement in E. unpaired t test, $^*P < 0.05$ versus control. Data expressed as mean ± SD in A-F (n = 6 in each group).

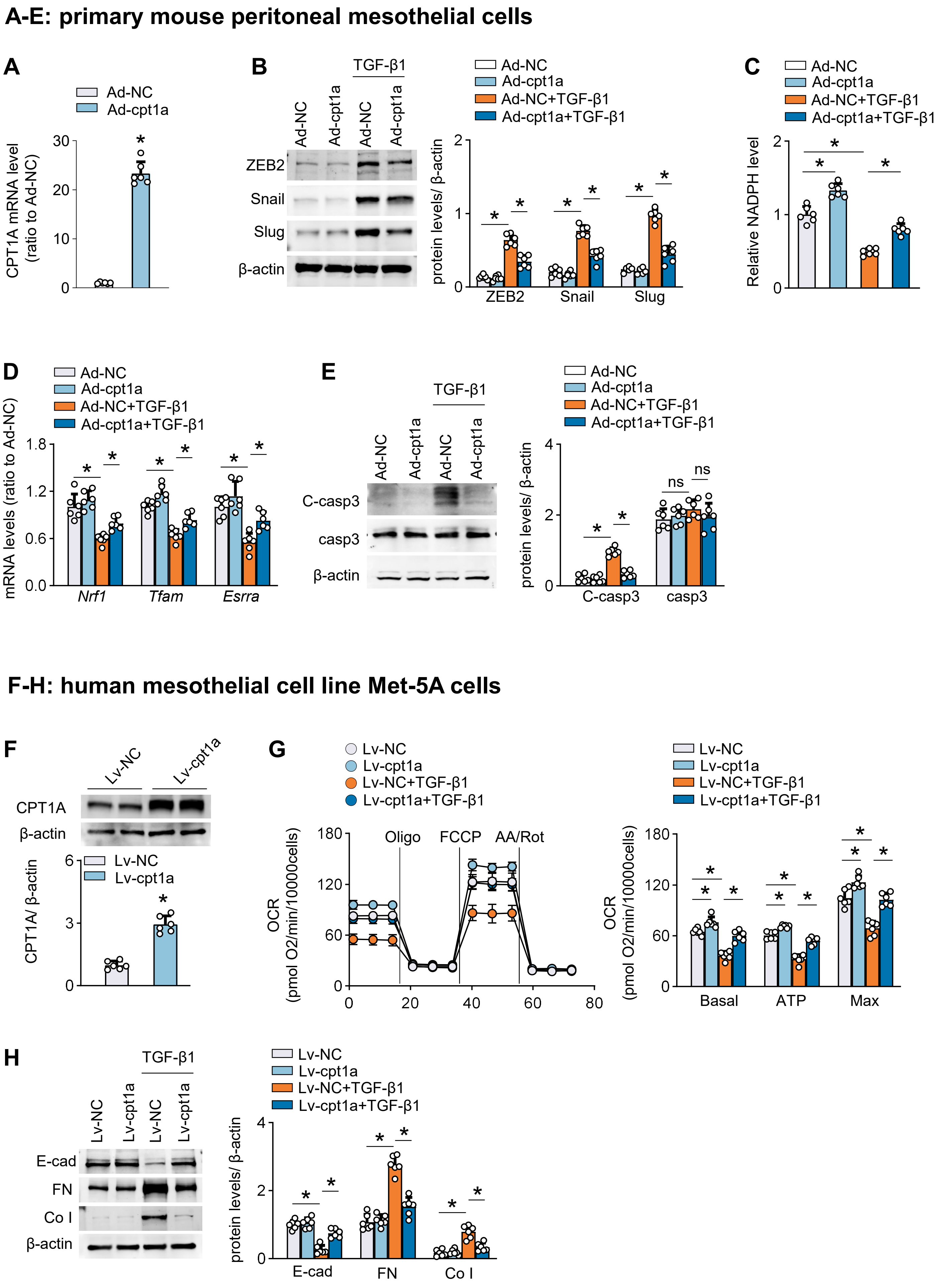
A-E: primary mouse peritoneal mesothelial cells
A
Ad-NC
Ad-cpt1a
CPT1A mRNA level (ratio to Ad-NC)
B
TGF-β1
Ad-NC
Ad-cpt1a
Ad-NC+TGF-β1
Ad-cpt1a+TGF-β1
ZEB2
Snail
Slug
β-actin
protein levels/ β-actin
C
Relative NADPH level
D
mRNA levels (ratio to Ad-NC)
Nrf1
Tfam
Esrra
E
C-casp3
casp3
ns
F-H: human mesothelial cell line Met-5A cells
F
Lv-NC
Lv-cpt1a
CPT1A
CPT1A/ β-actin
G
Lv-NC+TGF-β1
Lv-cpt1a+TGF-β1
Oligo
FCCP
AA/Rot
OCR (pmol O2/min/10000cells)
Basal
ATP
Max
H
E-cad
FN
Co I

Figure S6. Restoration of CPT1A expression reverses TGF-β1 induced FAO suppression and profibrotic phenotype in mesothelial cells

(A-E) Primary MPMCs are transfected with either Ad-CPT1A or Ad-NC. Real-time PCR showing upregulation of CPT1A in MPMCs transfected with Ad-CPT1A (A). Unpaired t test, $^*P < 0.05$ versus Ad-NC. MPMCs overexpressing CPT1A are protected from TGF-β1-induced detrimental changes, including levels of profibrogenic transcription factors (B), NADPH production (C), and expression of mitochondrial biogenesis-associated genes (D) and apoptosis marker C-casp3 (E). One-way ANOVA with Bonferroni correction, $^*P < 0.05$. (F-H) Met-5A cells are transfected with either Lv-CPT1A or Lv-NC. Immunoblots showing upregulation of CPT1A in Met-5A cells transfected with Lv-CPT1A (F). Unpaired t test, $^*P < 0.05$ versus Lv-NC. Overexpression of CPT1A in Met-5A cells reverses the ability of TGF-β1 to reduce OCR (G), and its ability to downregulate E-cad and upregulate FN and Co I (H). One-way ANOVA with Bonferroni correction, $^*P < 0.05$. Data expressed as mean ± SD in A-H ($n = 6$ in each group).

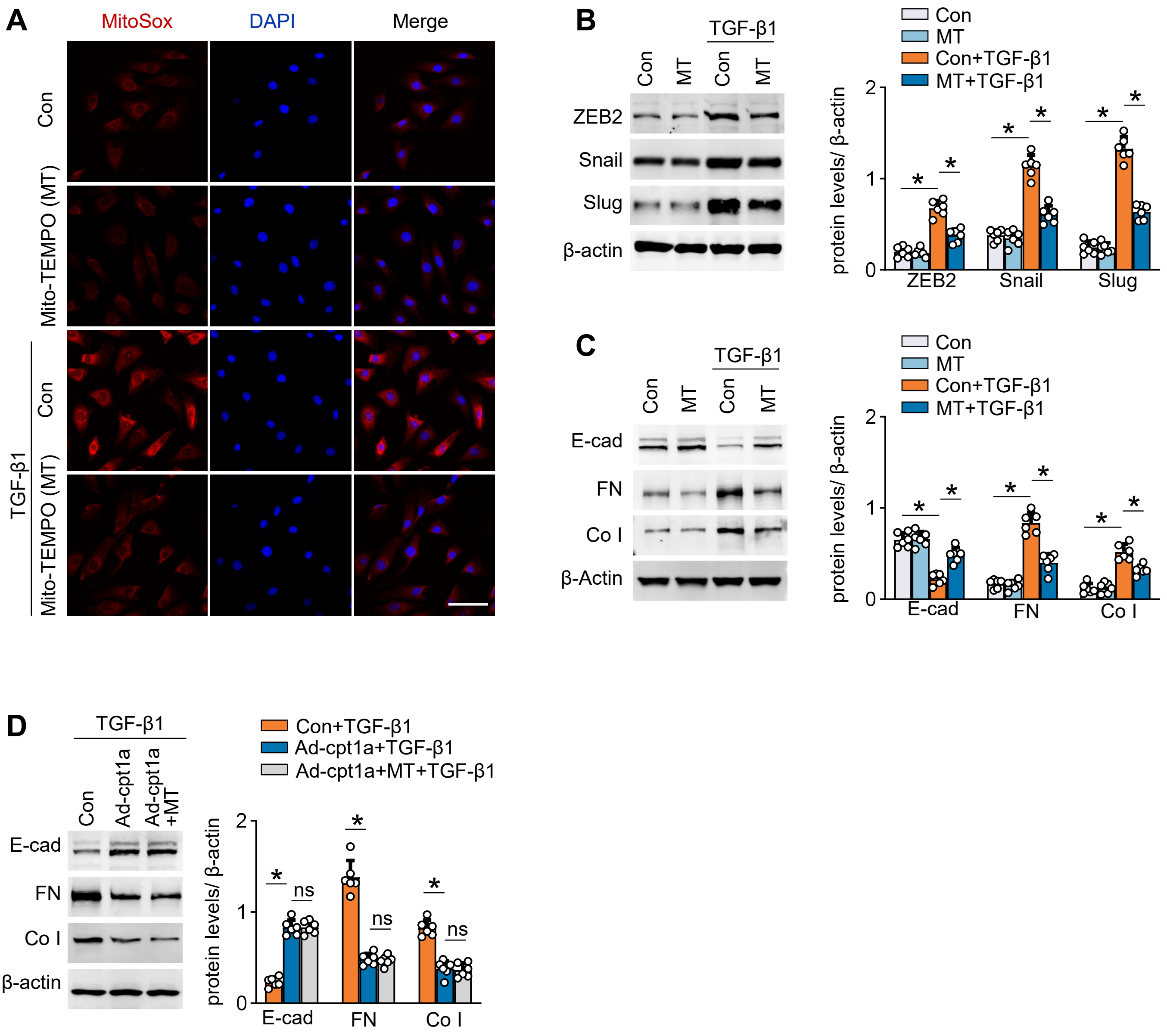

Figure S7. Mesothelial cells treated with Mito-TEMPO reverses TGF-β1-induced mitochondrial superoxide generation and profibrotic phenotype

(A-C) MPMCs treated with Mito-TEMPO are protected from TGF-β1-induced detrimental changes, including mitochondrial superoxide generation (A; Scale bar, 50 μm), and expression of profibrogenic transcription factors (B) and profibrotic markers (C). One-way ANOVA with Bonferroni correction, $*P < 0.05$. (D) CPT1A overexpression abolishes the protective responses induced by Mito-TEMPO in TGFβ1-treated MPMCs. One-way ANOVA with Bonferroni correction, $*P < 0.05$. ns, no significance. Data expressed as mean ± SD in B-D (n = 6 in each group).

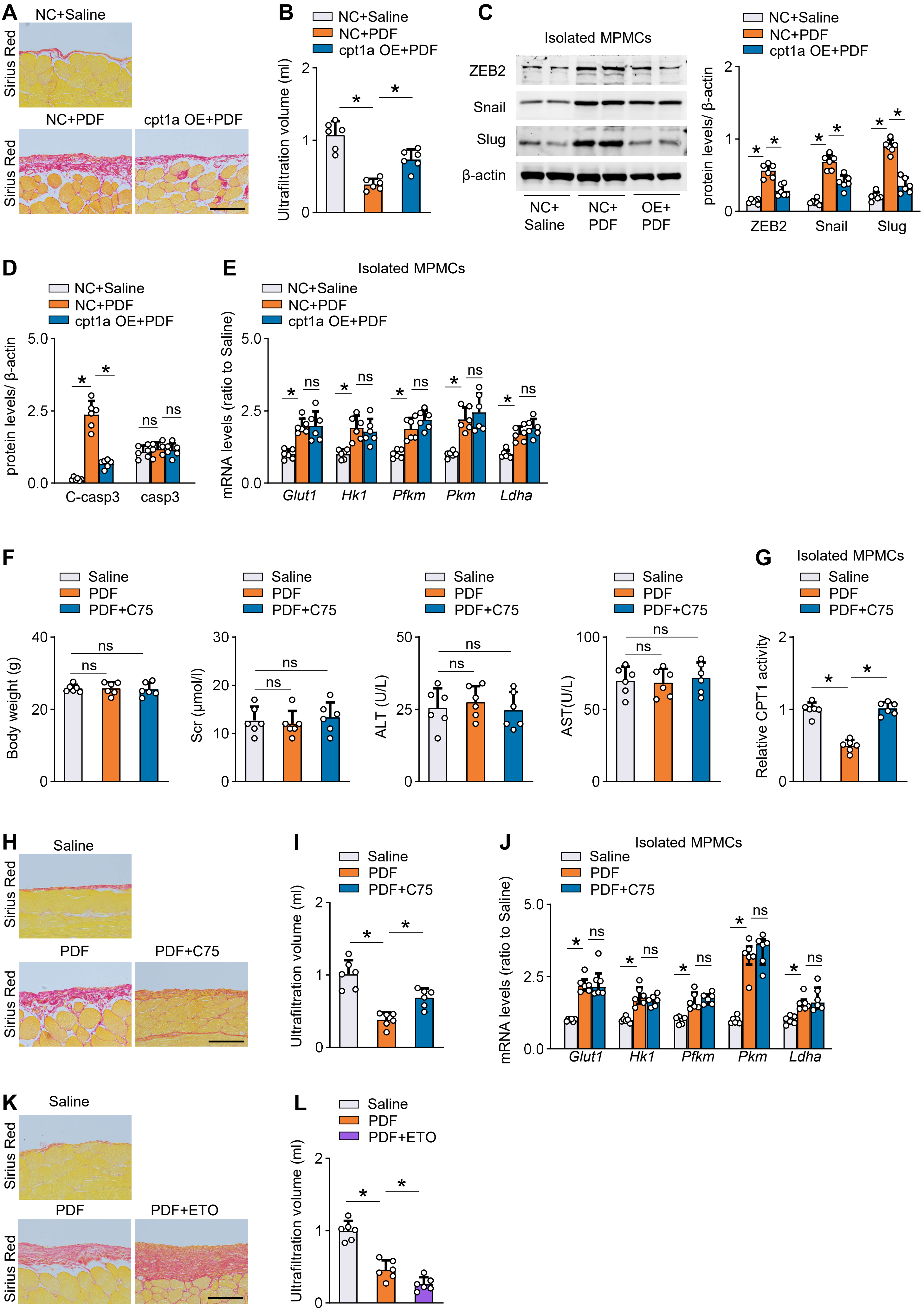
A
NC+Saline
NC+PDF
cpt1a OE+PDF
Sirius Red
B
NC+Saline
NC+PDF
cpt1a OE+PDF
Ultrafiltration volume (ml)
C
Isolated MPMCs
ZEB2
Snail
Slug
β-actin
NC+ Saline
NC+ PDF
OE+ PDF
protein levels/ β-actin
D
C-casp3
casp3
protein levels/ β-actin
E
Isolated MPMCs
mRNA levels (ratio to Saline)
Glut1
Hk1
Pfkm
Pkm
Ldha
F
Saline
PDF
PDF+C75
Body weight (g)
Scr (μmol/l)
ALT (U/L)
AST(U/L)
G
Isolated MPMCs
Relative CPT1 activity
H
Saline
PDF
PDF+C75
I
J
Isolated MPMCs
K
Saline
PDF
PDF+ETO
L

Figure S8. Restoration of CPT1A expression protects against fibrosis in mouse peritoneum

(A-E) Restoration of CPT1A in mesothelial cells in PD mice is achieved by injection of AAV-CPT1A-GFP (CPT1A OE) into peritoneal cavity of mice 21 days before daily PDF treatment. Peritoneal fibrosis presented by Sirius Red staining (A; Scale bar, 100 μm). The peritoneal ultrafiltration volume of mice examined by modified peritoneal equilibration test (B). Protein levels of profibrogenic transcription factors ZEB2, Snail and Slug in isolated MPMCs (C). Protein levels of C-casp3 and casp3 in isolated MPMCs (D). Transcript levels of glycolysis-related genes in isolated MPMCs (E). One-way ANOVA with Bonferroni correction, *P < 0.05. (F-J) Pharmacological improvement of FAO is achieved by intraperitoneal injection of mice with C75 three times per week starting 1 day after PDF treatment. Body weight, serum creatinine (Scr), Alanine aminotransferase (ALT) and Aspartate aminotransferase (AST) in mice (F). CPT1 activity in isolated MPMCs (G). Peritoneal fibrosis presented by Sirius Red staining (H; Scale bar, 100 μm). The peritoneal ultrafiltration volume of mice examined by modified peritoneal equilibration test (I). Transcript levels of glycolysis-related genes in isolated MPMCs (J). One-way ANOVA with Bonferroni correction in F, G and I; Kruskal-Wallis test in J, *P < 0.05. ns, no significance. (K and L) Pharmacological inhibition of FAO is achieved by intraperitoneally injecting mice with etomoxir three times per week starting 1 day after PDF treatment. Peritoneal fibrosis presented by Sirius Red staining. (K; Scale bar, 100 μm). The peritoneal ultrafiltration volume of mice examined by modified peritoneal equilibration test (L). One-way ANOVA with Bonferroni correction, *P < 0.05. Data expressed as mean ± SD in B-G, I and L; Data expressed as median with IQR in J (n = 6 in each group).

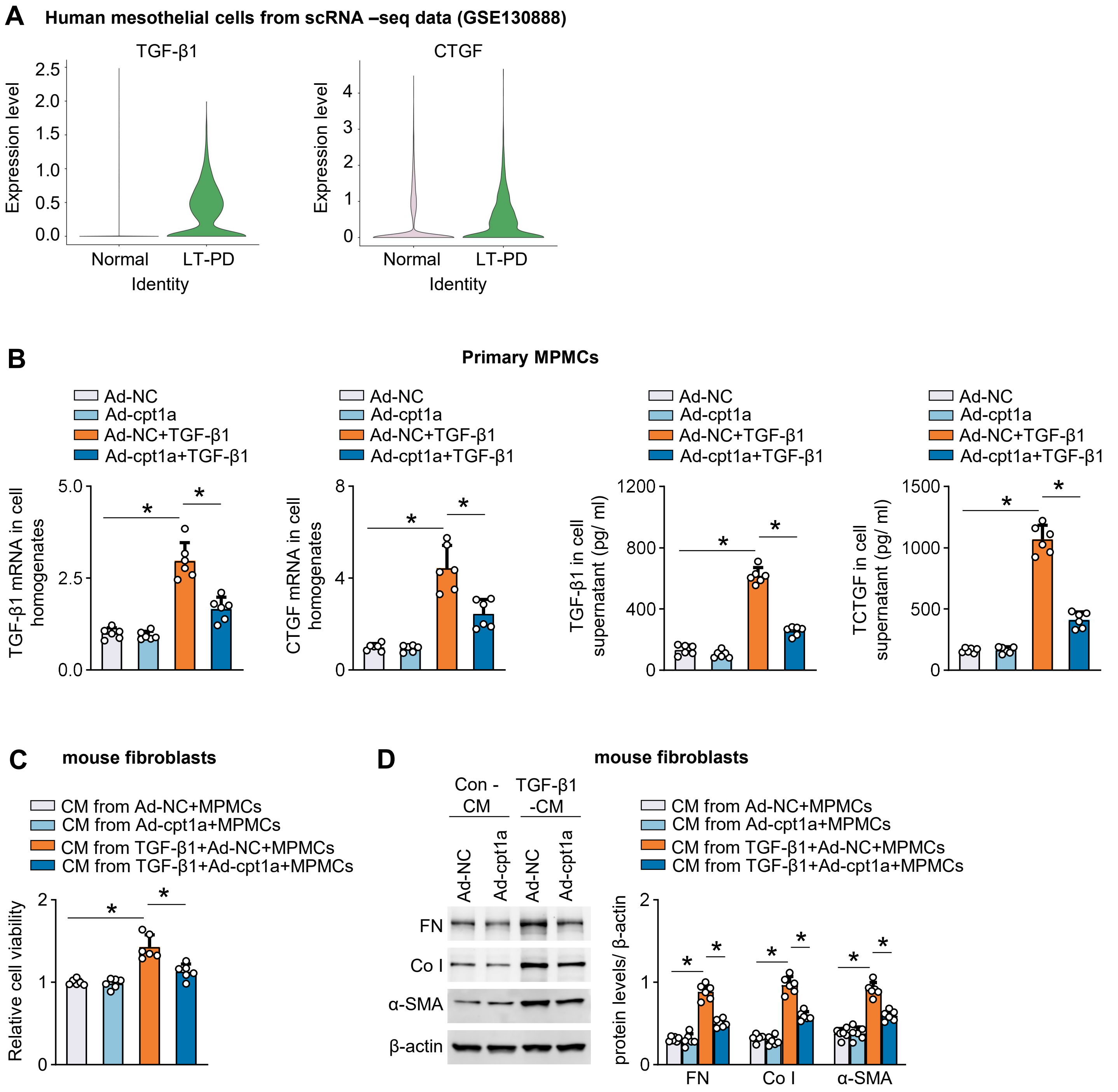

Figure S9. In the setting of PD, mesothelial cells display a profibrotic secretome that activates resident peritoneal fibroblasts

(A) Violin plots demonstrating the expression of TGF-β1 and CTGF in human mesothelial cells derived from the normal and LT-PD group in scRNA-seq data. (B-D) TGF-β1 treatment of primary MPMCs (5 ng/mL, 24 h) upregulates the expression of fibrogenic growth factors TGF-β1 and CTGF in both cell homogenates and supernatants (B). The conditioned medium from TGF-β1-treated mesothelial cells (TGF-β1-CM) enhances cell viability (C, measured by CCK-8 assay) and production of Fn, Co I and α-SMA (D) in fibroblasts. Transfection of MPMCs with Ad-CPT1A (versus Ad-NC) reverses all the TGF-β1-induced effects. One-way ANOVA with Bonferroni correction, *P < 0.05. Data expressed as mean ± SD in B-D (n = 6 in each group).
